# Universal linear intensity transformations using spatially incoherent diffractive processors

**DOI:** 10.1038/s41377-023-01234-y

**Published:** 2023-08-15

**Authors:** Md Sadman Sakib Rahman, Xilin Yang, Jingxi Li, Bijie Bai, Aydogan Ozcan

**Affiliations:** 1 Electrical and Computer Engineering Department, University of California, Los Angeles, CA 90095 USA; 2 Bioengineering Department, University of California, Los Angeles, CA 90095 USA; 3 California NanoSystems Institute (CNSI), University of California, Los Angeles, CA 90095 USA

**Keywords:** Imaging and sensing, Optical materials and structures

## Abstract

Under spatially coherent light, a diffractive optical network composed of structured surfaces can be designed to perform any arbitrary complex-valued linear transformation between its input and output fields-of-view (FOVs) if the total number (*N*) of optimizable phase-only diffractive features is ≥~2*N*_*i*_*N*_*o*_, where *N*_*i*_ and *N*_*o*_ refer to the number of useful pixels at the input and the output FOVs, respectively. Here we report the design of a spatially incoherent diffractive optical processor that can approximate any arbitrary linear transformation in time-averaged intensity between its input and output FOVs. Under spatially incoherent monochromatic light, the spatially varying intensity point spread function (*H*) of a diffractive network, corresponding to a given, arbitrarily-selected linear intensity transformation, can be written as *H*(*m*, *n*; *m*′, *n*′) = |*h*(*m*, *n*; *m*′, *n*′)|^2^, where *h* is the spatially coherent point spread function of the same diffractive network, and (*m*, *n*) and (*m*′, *n*′) define the coordinates of the output and input FOVs, respectively. Using numerical simulations and deep learning, supervised through examples of input-output profiles, we demonstrate that a spatially incoherent diffractive network can be trained to all-optically perform any arbitrary linear intensity transformation between its input and output if *N* ≥ ~2*N*_*i*_*N*_*o*_. We also report the design of spatially incoherent diffractive networks for linear processing of intensity information at multiple illumination wavelengths, operating simultaneously. Finally, we numerically demonstrate a diffractive network design that performs all-optical classification of handwritten digits under spatially incoherent illumination, achieving a test accuracy of >95%. Spatially incoherent diffractive networks will be broadly useful for designing all-optical visual processors that can work under natural light.

## Abstract (web summary)

Under spatially incoherent illumination, a diffractive optical processor with sufficient degrees of freedom (*N*) can perform any arbitrary linear intensity transformation between an input and an output field-of-view (FOV).

## Graphical abstract

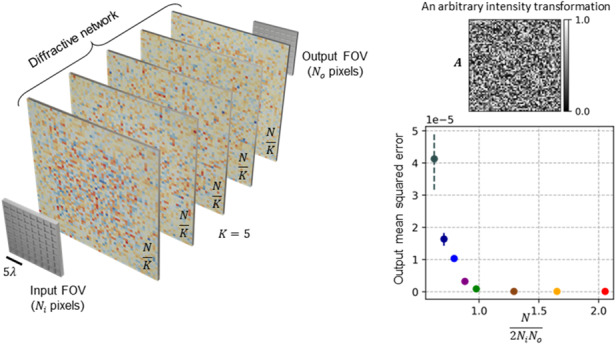

## Introduction

Spatial information processing with free-space optics has been widely explored and predates the proliferation of electronic computing^1–4^. Spatial filtering^5^, matrix multiplication^6–9^, Fourier transform^10,11^, implementation of neural networks^12,13^ and other information processing operations^14^ have been realized with free-space optics. The emergence of metasurfaces in the last decades, together with the search for neural network accelerators for artificial intelligence, has reignited the interest in free-space-based analog optical information processing^4,15–17^. The inherent transformation of optical fields as they propagate through free space, known as diffraction, together with the ability for wavefront modulation with compact hardware, makes low-cost and passive spatial information processing at the speed of light propagation possible^18,19^. Diffractive optics also enables the design of intricate optical elements and structures capable of shaping or controlling the light propagation for applications such as microscopy and imaging^20–24^. In recent years, diffractive optical networks comprising a set of spatially engineered surfaces to perform computation through passive light-matter-interaction have emerged as powerful all-optical processors^25,26^. Designed utilizing deep learning^27^, such coherent diffractive optical processors have demonstrated versatile applications, including statistical inference as well as deterministic tasks^26,28–34^ across the spectrum from terahertz to near-infrared^35^ and visible^36,37^.

Information processing with a diffractive network involves local modulation of the amplitude and/or the phase of the incident optical wave by structured surfaces containing diffractive neurons/features, each with a lateral size of ~*λ*/2, where λ is the wavelength of the spatially coherent illumination light. The entire propagation of a spatially coherent wave from the input plane to the output FOV comprises such optical modulations by *K* spatially optimized diffractive surfaces, which in total contain *N* independent diffractive features (for example, evenly distributed over the *K* diffractive surfaces). These *N* diffractive features represent the complex-valued transmission coefficients, forming the independent degrees of freedom of the diffractive processor, which can be optimized to all-optically execute different tasks^25,29–33,38,39^. It was shown that a spatially coherent diffractive optical network could be trained to perform any arbitrary complex-valued linear transformation between its input and output FOVs if *N* ≥ *N*_*i*_*N*_*o*_, where *N*_*i*_ and *N*_*o*_ refer to the number of useful (diffraction-limited) pixels at the input and the output FOVs^19^. For a phase-only diffractive network where the transmission coefficients of the diffractive features of each structured surface only modulate the phase information of light, the requirement for universal linear transformations increases to *N* ≥ 2*N*_*i*_*N*_*o*_ due to the reduced degrees of freedom that can be optimized independently.

For a given complex-valued linear transformation that a coherent diffractive network is designed to approximate, any arbitrary point on the input plane defined by (*m*′, *n*′) will result in a unique complex-valued coherent point spread function (*h*) at the output FOV defined by (*m*, *n*). This 4-dimensional complex-valued function, *h*(*m*, *n*; *m*′, *n*′), that maps the input and output FOVs represents a *spatially varying* coherent point spread function (PSF). Stated differently, unlike traditional spatially invariant imaging systems, a coherent diffractive optical network provides a framework to approximate any arbitrary *h*(*m*, *n*; *m*′, *n*′) that corresponds to an arbitrarily selected complex-valued linear transformation between its input and output FOVs. It was also shown that different/independent complex-valued linear transformations could be multiplexed in a single spatially coherent diffractive processor by utilizing polarization and wavelength diversity^40,41^.

All of these earlier studies on universal linear transformations implemented in free space through diffractive processors were based on spatially coherent illumination. In this paper, we report the demonstration of universal linear transformations in optical intensity performed under spatially incoherent monochromatic illumination of an input FOV. Using numerical simulations, we show that, under spatially incoherent light, a diffractive optical processor can perform any arbitrary linear transformation of time-averaged intensities between its input and the output FOVs. Our numerical analyses revealed that phase-only diffractive optical processors with a shallow architecture (for example, having a single trainable diffractive surface) are unable to accurately approximate an arbitrary intensity transformation irrespective of the total number (*N*) of diffractive features available for optimization; on the contrary, phase-only diffractive optical processors with deeper architectures (one diffractive layer following others) can perform an arbitrary intensity linear transformation using spatially incoherent illumination with a negligible error when *N* ≥ 2*N*_*i*_*N*_*o*_. We also demonstrate that spatially incoherent diffractive optical processors can perform linear intensity transformations at different illumination wavelengths, i.e., simultaneously perform the same linear transformation or different linear transformations at different wavelengths under spatially incoherent illumination. Finally, we report the design of a spatially incoherent diffractive network for all-optical classification of handwritten digits, achieving 95.04% blind testing accuracy.

These analyses and conclusions are important for all-optical information processing and visual computing systems that use spatially and temporally incoherent light, such as in natural scenes. The presented framework can also find unique applications in computational microscopy and incoherent imaging through point spread function engineering.

## Results

In this paper, we use the terms “diffractive optical network”, “diffractive optical processor”, “diffractive network” and “diffractive processor” interchangeably. Similarly, the terms “diffractive surface” and “diffractive layer” are used interchangeably. In the next sub-section, we start with a theoretical analysis of spatially incoherent diffractive optical processing of visual information.

### Theoretical analysis

Spatially coherent monochromatic diffractive optical networks can be characterized by a 4-dimensional complex-valued coherent impulse response function (i.e., the point spread function) that is spatially varying, connecting the input and output FOVs: *h*(*x*, *y*; *x*′, *y*′). Stated differently, each arbitrarily selected complex-valued linear transformation that is desired between the pixels of an input FOV and output FOV results in a spatially varying impulse response function *h*(*x*, *y*; *x*′, *y*′), where (*x*′, *y*′) and (*x*, *y*) define the input and output FOVs, respectively. Based on this definition, the complex-valued output field *o*_*c*_(*x*, *y*) of a spatially coherent diffractive processor is related to the complex-valued input field *i*_*c*_(*x*′, *y*′) by:

1 $${o}_{c}\left(x,y\right)=\iint {h}_{c}\left(x,y{\rm{;}}\,{x}^{{\prime} },{y}^{{\prime} }\right){i}_{c}\left({x}^{{\prime} },{y}^{{\prime} }\right)d{x}^{{\prime} }d{y}^{{\prime} }$$

The subscript *c* indicates that the quantities are functions of continuous spatial variables *x*, *y*, *x*′, *y*′, representing the transverse coordinates on the output and input planes. If these optical fields are sampled at an interval (*δ*) sufficiently small to preserve the spatial variations, satisfying the Nyquist criterion^42^, one can write:

2 $$o\left(m,n\right)=\sum _{{m}^{{\prime} },{n}^{{\prime} }}h\left(m,n{\rm{;}}\,{m}^{{\prime} },{n}^{{\prime} }\right)\,i\left({m}^{{\prime} },{n}^{{\prime} }\right)$$

Here, *m*, *n*, *m*′, *n*′ refer to discrete indices such that *o*(*m, n*) = *o*_*c*_(*mδ*, *nδ*) and *i*(*m*′, *n*′) = *i*_*c*_(*m*′*δ*, *n*′*δ*). The instantaneous output intensity can be written as:

3 $$\begin{array}{c}{\left|o\left(m,n\right)\right|}^{2}=\sum\limits_{{m}^{{\prime} },{n}^{{\prime} },{m}^{{''}},{n}^{''}}h\left(m,n;\,{m}^{{\prime}},{n}^{{\prime}}\right)\,{h}^{*}\left(m,n;\,{m}^{''},{n}^{''}\right)\,\left|i\left({m}^{{\prime} },{n}^{{\prime}}\right)\right|\,\left|i\left({m}^{''},{n}^{''}\right)\right|\,{e}^{j\left(\varphi \left({m}^{{\prime} },{n}^{{\prime} }\right)-\varphi\left({m}^{''},{n}^{''}\right)\right)}\end{array}$$

where *φ*(.) is the phase function of the input field *i*, i.e., *i* = |*i*|*e*^*jφ*^, and h^*^ denotes the complex conjugate of *h*. The time-averaged output intensity can be written as:

4 $$\begin{array}{c}O\left(m,n\right)=\left\langle {\left|o\left(m,n\right)\right|}^{2}\right\rangle =\sum\limits_{{m}^{{\prime} },{n}^{{\prime} },{m}^{''},{n}^{''}}h\left(m,n;\,{m}^{{\prime} },{n}^{{\prime}}\right)\,{h}^{*}\left(m,n;\,{m}^{''},{n}^{''}\right)\left|i\left({m}^{{\prime} },{n}^{{\prime} }\right)\right|\left|i\left({m}^{''},{n}^{''}\right)\right|\left\langle {e}^{j\Delta \varphi }\right\rangle \end{array}$$

where 〈·〉 denotes time-average operation and ∆*φ* = *φ*(*m*′, *n*′) − *φ*(*m*″, *n*″). Since the illumination light is spatially incoherent, the phases at different spatial points of the input vary randomly over time and are independent of each other^43^. Stated differently, for stationary objects/scenes that are uniformly illuminated with a spatially incoherent light, ∆*φ* varies randomly between 0 and 2*π* over time, yielding 〈*e*^*j*∆*φ*^〉 = 0 for (*m*′, *n*′) ≠ (*m″*, *n*″). As a result of this, under spatially incoherent illumination, Eq. (4) can be written as:

5 $$O\left(m,n\right)=\sum _{{m}^{{\prime} },{n}^{{\prime} }}{\left|h\left(m,n{\rm{;}}\,{m}^{{\prime} },{n}^{{\prime} }\right)\right|}^{2}\,\left\langle {\left|i\left({m}^{{\prime} },{n}^{{\prime} }\right)\right|}^{2}\right\rangle =\sum _{{m}^{{\prime} },{n}^{{\prime} }}H\left(m,n{\rm{;}}\,{m}^{{\prime} },{n}^{{\prime} }\right)\,I\left({m}^{{\prime} },{n}^{{\prime} }\right)$$

where *I* = 〈|*i*|^2^〉 is the time-averaged input intensity and *H*(*m*, *n*; *m*′, *n*′) = |*h*(*m*, *n*; *m*′, *n*′)|^2^ is the intensity impulse response of the diffractive optical processor under spatially incoherent illumination. From now on, unless otherwise stated, we use the term optical “intensity” to imply time-averaged intensity functions. Similarly, whenever all-optical linear transformation of intensity is mentioned, spatially incoherent monochromatic illumination is implied unless stated otherwise.

We should emphasize that while *H*(*m*, *n*; *m*′, *n*′) = |*h*(*m*, *n*; *m*′, *n*′)|^2^, we have in general *O*(*m, n*) ≠ |*o*(*m*, *n*)|^2^. Therefore, the output intensity of a spatially incoherent diffractive network cannot be calculated as |*o*(*m, n*)|^2^ = |∑_*m*′,*n*′_ *h*(*m, n; m*′, *n*′) *i*(*m*′, *n*′)|^2^. For the numerical forward model corresponding to each input object, as will be detailed in the next section, we used a large number of random phase distributions at the input plane to approximate *O*(*m, n*) = 〈|*o*(*m, n*)|^2^〉 under spatially incoherent illumination.

### Numerical analysis

**Fig. 1:**
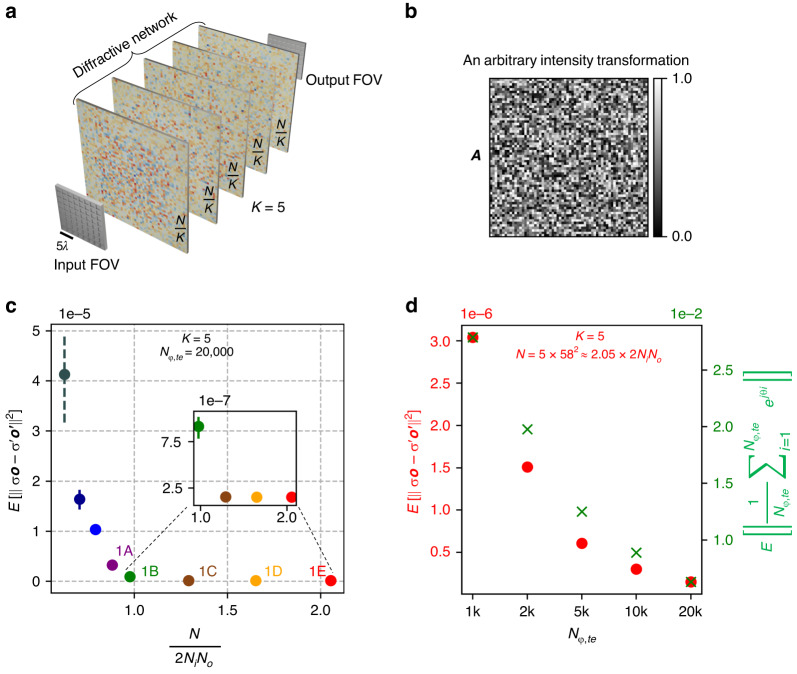
All-optical linear transformation of intensity performed by diffractive networks under spatially incoherent illumination. **a** Schematic of a diffractive network formed by *K* = 5 diffractive surfaces that all-optically perform a linear transformation of intensity between the input and output FOVs. The *N* diffractive features are distributed evenly among the *K* = 5 surfaces. **b** An arbitrary *N*_*o*_ × *N*_*i*_ matrix ***A***, representing the target intensity transformation to be performed all-optically by the diffractive network. Here *N*_*i*_ = 8^2^ and *N*_*o*_ = 8^2^ are the number of pixels at the input and the output FOVs of the diffractive network, respectively. **c** The expectation value of the MSE between the all-optical output intensity ***o***′ and the ground-truth output intensity ***o***, as a function of *N* for different diffractive networks trained using the *indirect* approach. To simulate the incoherent propagation of intensity for each test input, we used *N*_*φ,te*_ = 20,000. **d** Dependence of the calculated output MSE on *N*_*φ,te*_, demonstrated for network # 1E of Fig. 1c. The right y-axis shows the expectation value of the residual magnitude of $\frac{1}{{N}_{\varphi ,{te}}}{\sum }_{i=1}^{{N}_{\varphi ,{te}}}{e}^{j{\theta }_{i}}$, where *θ*_*i*_ ~ *Uniform*(0, 2*π*)

In this subsection, we numerically explore the design of diffractive optical processors to perform an arbitrary linear intensity transformation between the input and the output FOVs under spatially incoherent illumination. We assume, as shown in Fig. 1a, *N* independent diffractive features (phase-only elements) that are distributed over *K* diffractive surfaces, each with *N*/*K* diffractive features, between the input and output planes. Following from Eq. (5), if we rearrange the pixel intensities of *I*(*m*′, *n*′) and *O*(*m*, *n*) as column vectors ***i*** and ***o***, then we can write ***o*** = ***A***′ ***i***, where ***A***′ represents the linear intensity transformation performed by the diffractive optical network under spatially incoherent illumination. The elements of ***A***′ correspond to the elements of the intensity impulse response *H*(*m*, *n*; *m*′, *n*′); see Eq. (5). Note that all the elements of ***A***′ are real and nonnegative since it represents a linear intensity transformation with *H*(*m, n; m*′, *n*′) = |*h*(*m, n; m'*, *n'*)|^2^. Hence, in the context of arbitrary linear transformations in intensity, only real transformation matrices with nonnegative elements are considered.

For our target linear transformation that is to be approximated by the spatially incoherent diffractive processor, initially, we selected an arbitrary matrix ***A***, as shown in Fig. 1b. In the following numerical analysis, we optimize *N* diffractive features of a phase-only diffractive processor so that ***A***′ ≈ ***A*** under spatially incoherent illumination. The size of ***A*** is chosen as *N*_*o*_ × *N*_*i*_ = 64 × 64, i.e., the number of pixels at both the input (*N*_*i*_) and the output (*N*_*o*_) FOVs are 8 × 8, arranged in a square grid. Each element of the matrix ***A*** is randomly sampled from a uniform probability distribution between 0 and 1, i.e., ***A***[*p, q*] ~ *Uniform*(0, 1) where ***A***[*p, q*] is the element at *p*-th row and *q*-th column of ***A***, *p* = 1,…,*N*_*o*_ and *q* = 1,…,*N*_*i*_.

For the deep learning-based optimization of the design of a phase-only diffractive processor to achieve ***A***′ ≈ ***A***, we followed two different ***data-driven supervised learning*** approaches: (1) *the indirect approach* and (2) *the direct approach*. In the indirect approach, instead of directly training the diffractive network to perform the linear intensity transformation ***A***, we trained the network, under *spatially coherent* illumination, to perform the complex-valued linear transformation $\overline{\overline{{\boldsymbol{A}}}}$ between the input and output FOVs such that $|\overline{\overline{{\boldsymbol{A}}}}[p,q]|=\sqrt{{\boldsymbol{A}}[p,q]}$, which would result in an intensity linear transformation ${|\overline{\overline{{\boldsymbol{A}}}}[p,q]|}^{2}={\boldsymbol{A}}[p,q]$ under spatially incoherent illumination. For the purpose of the training, we defined the phase of $\overline{\overline{{\boldsymbol{A}}}}[p,q]$ to be zero, i.e., $\overline{\overline{{\boldsymbol{A}}}}[p,q]=\sqrt{{\boldsymbol{A}}[p,q]}\,{\exp}\left({j0}\right)$; however, any other phase distribution could also be used since the design space is not unique. Stated differently, in this indirect approach, we design a diffractive network that can achieve a spatially coherent impulse response *h*(*m, n; m*′, *n*′), which will ensure that the same design has a spatially incoherent impulse response of *H*(*m, n; m*′, *n*′) = |*h*(*m, n; m*′, *n*′)|^2^ such that ***A***′ ≈ ***A*** can be satisfied under spatially incoherent illumination. To achieve this goal, we used the relationship $\tilde{{\boldsymbol{o}}}=\overline{\overline{\boldsymbol{A}}}\,\tilde{\boldsymbol{i}}$ to generate a large set of input-target complex-valued optical field pairs $\left(\widetilde{{\boldsymbol{i}}},\widetilde{{\boldsymbol{o}}}\right)$, and used deep learning to optimize the phase values of the diffractive features by minimizing the mean squared error (MSE) loss between the target complex field $\widetilde{{\boldsymbol{o}}}$ and the complex field $\widetilde{{\boldsymbol{o}}}^{\prime}$ obtained by coherently propagating $\widetilde{{\boldsymbol{i}}}$ through the diffractive network (see the “Materials and methods” section). In other words, spatially coherent design of a diffractive network is used here as a proxy for the design of a spatially incoherent diffractive network that can achieve any arbitrary intensity linear transformation between its input and output FOVs.

In the second approach (termed the direct approach), we trained the diffractive network to perform the desired intensity linear transformation ***A*** between the input and the output FOVs, by directly using the relationship ***o*** = ***Ai*** to generate a large set of input-target intensity pairs **(*i***, ***o*)**. Using this large training set of input/output intensity patterns, we optimized the transmission phase values of the diffractive layers using deep learning, by minimizing the MSE loss between the output pixel intensities of the diffractive processor ***o***′ and the ground-truth intensities ***o*** (see the “Materials and methods” section). During the training phase, the output intensity of the diffractive processor was simulated through the incoherent propagation of the input intensity patterns, ***i*** or *I*(*m*′, *n*′). To numerically simulate the spatially incoherent propagation of *I*(*m*′, *n*′), we assumed the input optical field to be $\sqrt{I}{e}^{j\varphi }$ where *φ* is a random 2D phase distribution, i.e., *φ*(*m*′, *n*′) ~ *Uniform*(0, 2*π*) for each (*m*′, *n*′). This input field with the random phase distribution *φ* was coherently propagated through the diffractive surfaces to the output plane, using the angular spectrum approach^25^. We repeated this coherent wave propagation *N*_*φ*_ times for every ***i***, each time with a different random phase *φ*(*m*′, *n*′) distribution, and averaged the resulting *N*_*φ*_ output intensities. As *N*_*φ*_ → ∞, the average intensity would approach the theoretical time-averaged output intensity for spatially incoherent illumination, i.e., *O*(*m, n*) = 〈|*o*(*m, n*)|^2^〉. Due to the limited availability of computational resources, for the direct training (the second design approach) of the spatially incoherent diffractive optical processors, we used *N*_*φ*_ = *N*_*φ,tr*_ = 1000.

**Fig. 2:**
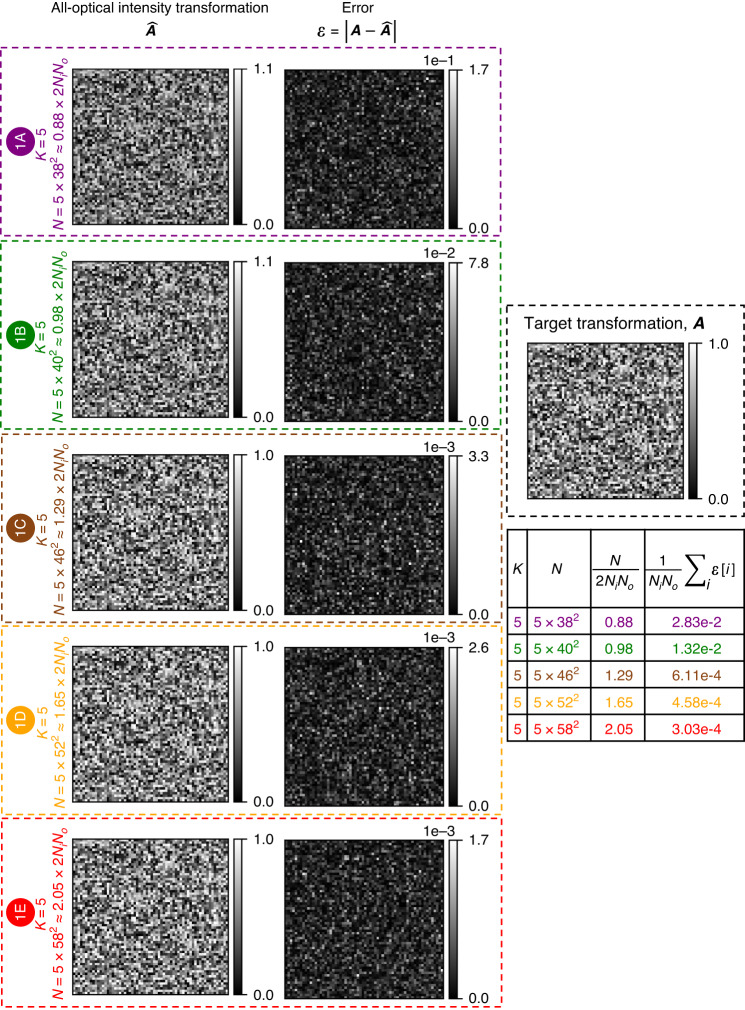
All-optical intensity linear transformations under spatially incoherent illumination performed by diffractive networks trained using the *indirect* approach. All-optical linear transformations of intensity, $\hat{{\boldsymbol{A}}}$, performed under spatially incoherent illumination, by five of the diffractive network designs shown in Fig. 1c, together with the corresponding error matrices with respect to the target transformation, ${\boldsymbol{\varepsilon }}=\left|{\boldsymbol{A}}-\hat{{\boldsymbol{A}}}\right|$. Here |**∙**| denotes elementwise operation. The means of the error matrix elements are listed in the table on the right

**Fig. 3:**
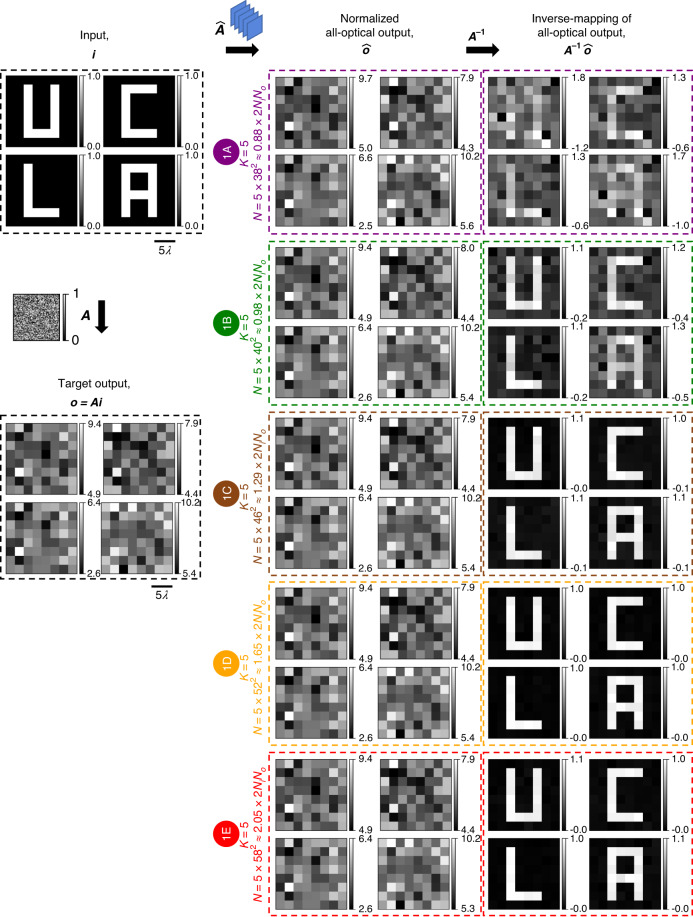
All-optical linear transformations of test intensity patterns under spatially incoherent illumination performed by diffractive networks trained using the *indirect* approach. All-optical linear transformation of structured intensity patterns such as letters U, C, L, and A by the same diffractive networks as in Fig. 2, accompanied by the patterns resulting from the numerical inverse mapping of the all-optical outputs through multiplication by ***A***^**−1**^

**Fig. 4:**
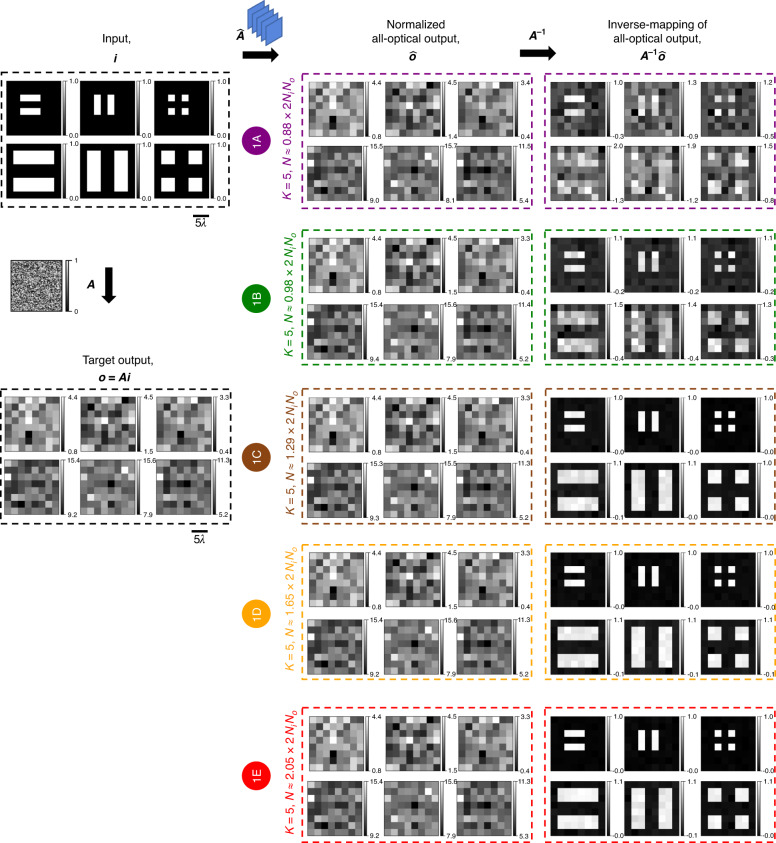
All-optical linear transformations of test intensity patterns under spatially incoherent illumination performed by diffractive networks trained using the *indirect* approach. Same as Fig. 3, except for the test intensity patterns formed by closely separated lines and points

**Fig. 5:**
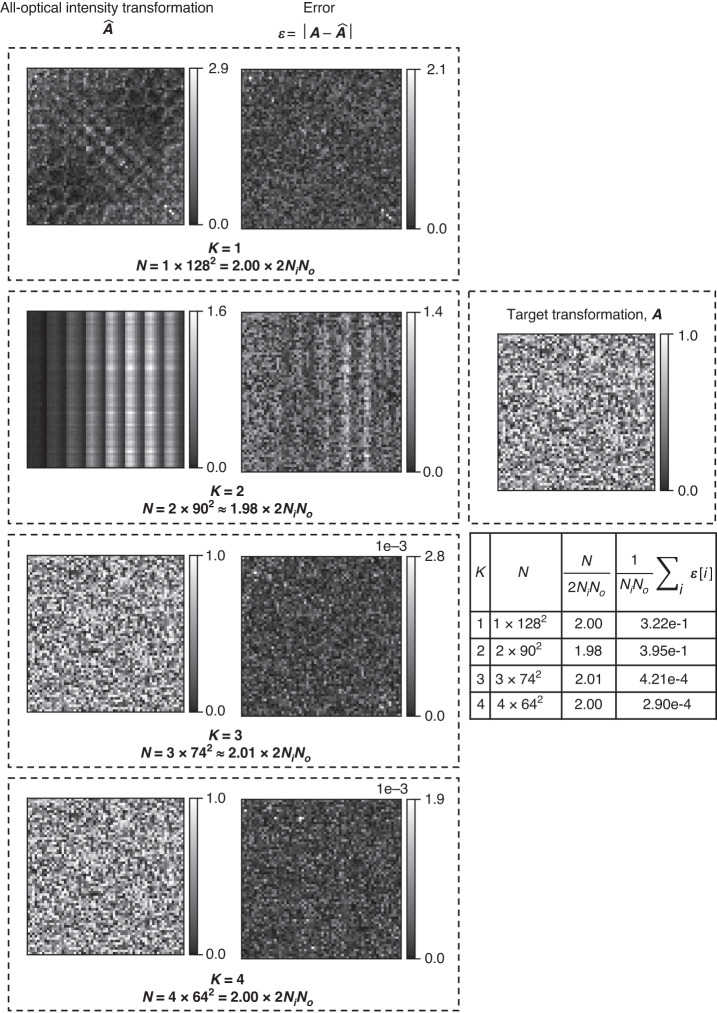
Effect of the diffractive network’s depth, i.e., the number of diffractive surfaces (*K*), on the approximation performance for an arbitrary intensity linear transformation under spatially incoherent illumination. All-optical linear transformations of intensity, $\hat{{\boldsymbol{A}}}$, performed by four diffractive network designs with approximately equal *N* and increasing *K*, are shown, together with the corresponding error matrices with respect to the target transformation, i.e., ${\boldsymbol{\varepsilon }}=\left|{\boldsymbol{A}}-\hat{{\boldsymbol{A}}}\right|$. Here |**∙**| denotes elementwise operation. The mean values of the error matrix elements are listed in the table on the right

**Fig. 6:**
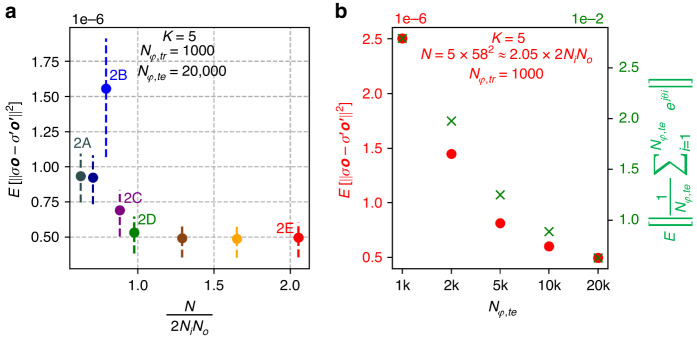
All-optical linear transformation of intensity under spatially incoherent illumination, by diffractive networks trained using the direct approach. **a** The expectation value of the MSE between the all-optical output intensity ***o***′ and the ground-truth output intensity ***o***, as a function of *N* for different diffractive networks trained using the direct approach. **b** Dependence of the calculated output MSE on *N*_*φ,te*_, demonstrated for network # 2E of Fig. 6a. The right y-axis shows the expectation value of the residual magnitude of $\frac{1}{{N}_{\varphi ,{te}}}{\sum }_{i=1}^{{N}_{\varphi ,{te}}}{e}^{j{\theta }_{i}}$, where *θ*_*i*_ ~ *Uniform*(0, 2*π*)

**Fig. 7:**
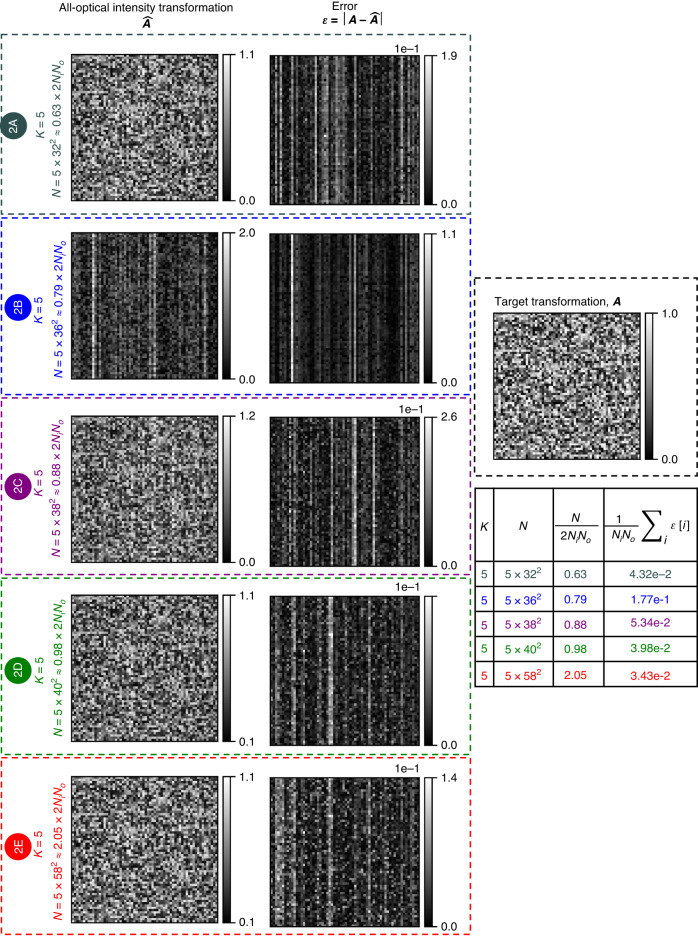
All-optical intensity linear transformations under spatially incoherent illumination performed by diffractive networks trained using the *direct* approach. All-optical linear transformations of intensity, $\hat{{\boldsymbol{A}}}$, performed under spatially incoherent illumination by five of the diffractive network designs shown in Fig. 6a, together with the corresponding error matrices with respect to the target transformation, ${\boldsymbol{\varepsilon }}=\left|{\boldsymbol{A}}-\hat{{\boldsymbol{A}}}\right|$. Here |**∙**| denotes elementwise operation. The mean values of the error matrix elements are listed in the table on the right

**Fig. 8:**
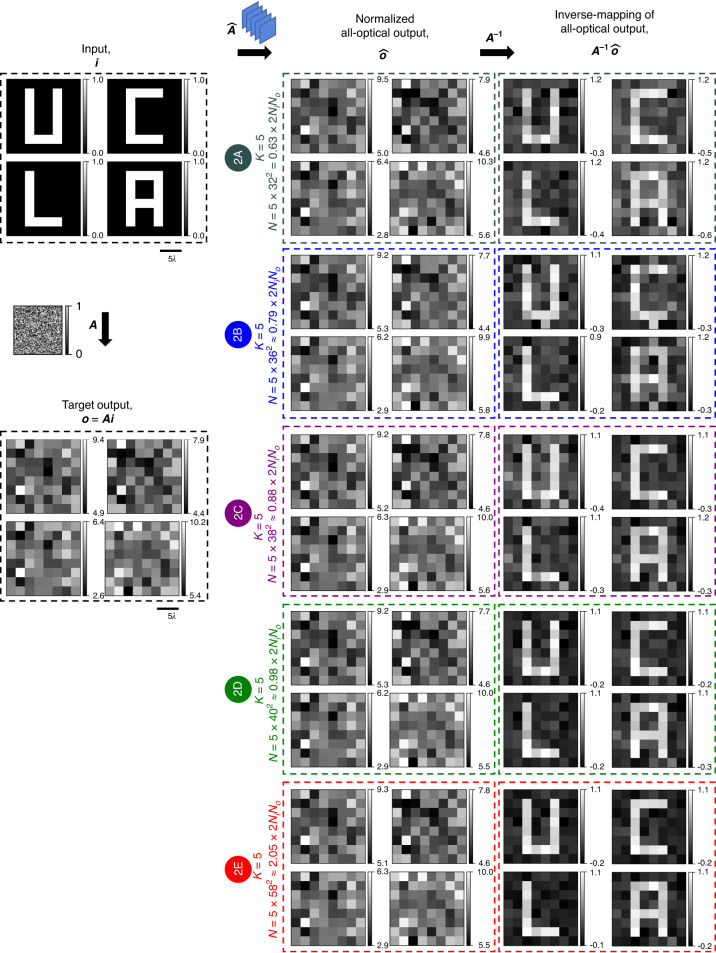
All-optical linear transformations of test intensity patterns under spatially incoherent illumination performed by diffractive networks trained using the *direct* approach. All-optical linear transformation of structured intensity patterns such as letters U, C, L, and A by the same diffractive networks as in Fig. 7, accompanied by the patterns resulting from the numerical inverse mapping of the all-optical outputs through multiplication by ***A***^**−1**^

**Fig. 9:**
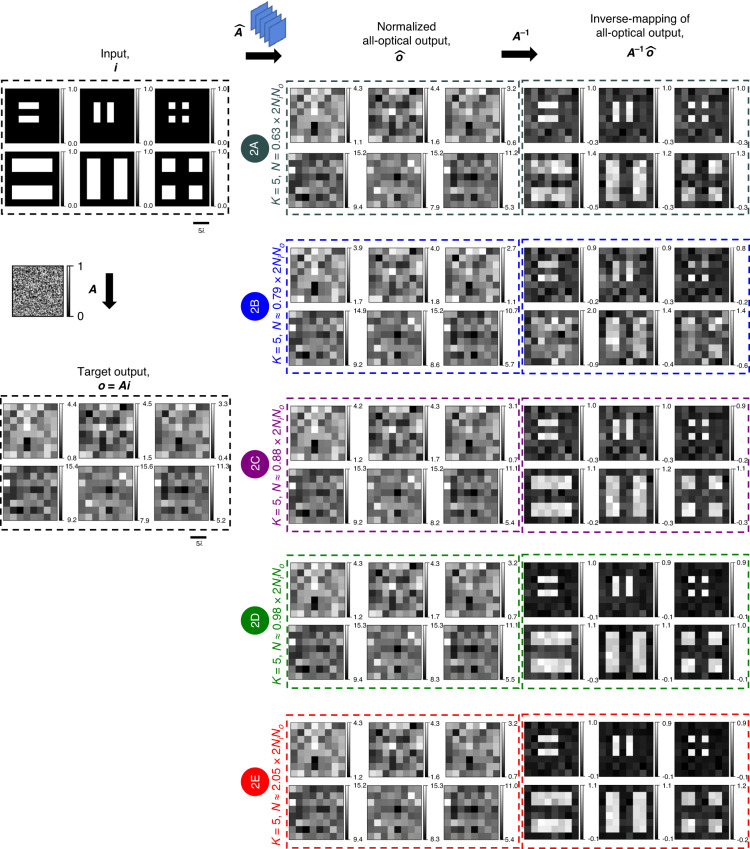
All-optical linear transformations of test intensity patterns under spatially incoherent illumination performed by diffractive networks trained using the *direct* approach. Same as Fig. 8, except for the test intensity patterns formed by closely separated lines and points

**Fig. 10:**
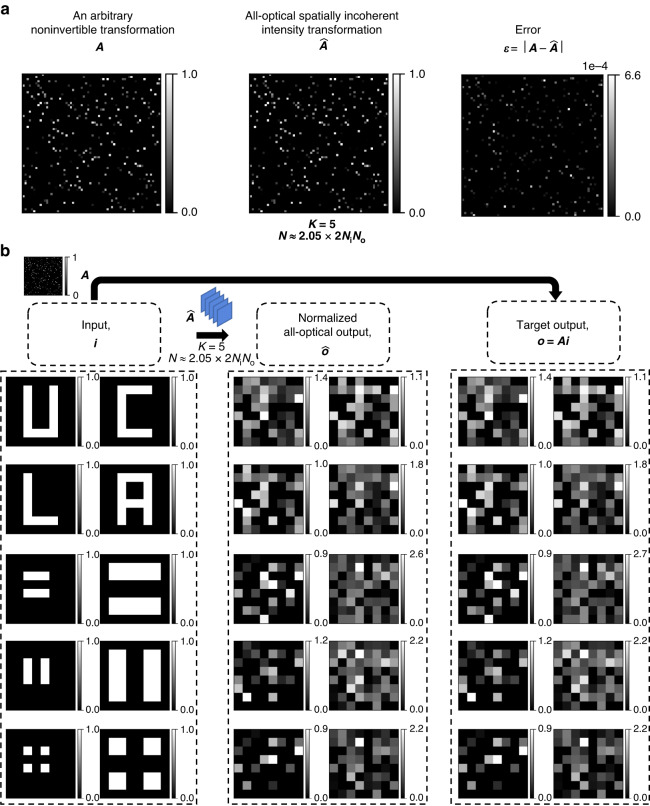
Approximation of an arbitrary non-invertible linear transformation (*A*) of intensity, under spatially incoherent illumination, by a diffractive network (*K* = 5, *N* = 5 × 58^2^) trained using the indirect approach. **a** The target transformation ***A***, the all-optical intensity transformation $\hat{{\boldsymbol{A}}}$ performed by the trained diffractive network and the error matrix ${\boldsymbol{\varepsilon }}=\left|{\boldsymbol{A}}-\hat{{\boldsymbol{A}}}\right|$. Here |**∙**| denotes elementwise operation. **b** All-optical transformation of different test intensity patterns by the trained diffractive network, together with the corresponding ground truths

The diffractive models reported in Figs. 1–5 and 10 were trained using the indirect approach while the ones in Figs. 6–9 were trained using the direct approach. All the diffractive networks reported in this work, after their training using either the direct or indirect design approaches, were evaluated and blindly tested through the incoherent propagation of input intensities with *N*_*φ,te*_ = 20,000. Since the testing is computationally less cumbersome compared to the training, we used *N*_*φ,te*_ = 20,000 ≫ *N*_*φ,tr*_.

Unless otherwise stated, we assumed the size of the input and the output pixels to be ~2.13*λ* × 2.13*λ*, where *λ* is the illumination wavelength. After the training phase, we tested the resulting diffractive processor designs using 20,000 test intensity patterns ***i*** that were never used during training; the size of this testing intensity set (20,000) should not be confused with *N*_*φ,te*_ = 20,000 since for each input intensity test pattern of this set, we used *N*_*φ,te*_ = 20,000 random 2D phase patterns to compute the corresponding spatially incoherent output intensity. In Fig. 1c, the approximation errors of eight different phase-only diffractive processors trained using the indirect approach, each with *K* = 5 diffractive layers, are reported as a function of *N*. The mean error (Fig. 1c) for each diffractive design was calculated at the output intensity patterns ***o***′ with respect to the ground truth ***o*** = ***Ai***, by averaging over the 20,000 test intensity patterns. Fig. 1c reveals that the approximation error of the spatially incoherent diffractive processors reaches a minimum level as $\tfrac{N}{2{N}_{i}{N}_{o}}$ approaches 1, and stays at the same level for *N* ≥ 2*N*_*i*_*N*_*o*_.

To understand the impact of *N*_*φ,te*_ on these approximation error calculations, we took the diffractive processor design # 1E shown in Fig. 1c (i.e., *K* = 5, *N* ≈ 2.1 × 2*N*_*i*_*N*_*o*_), and used different *N*_*φ,te*_ values at the blind testing phase for evaluating the average test error on the same intensity test set composed of 20,000 patterns ***i***. As shown in Fig. 1d, the computed error values decrease as *N*_*φ,te*_ increases, as expected. On the right y-axis of the same Fig. 1d, we also show, as a function of *N*_*φ,te*_, the expectation value of $\left|\tfrac{1}{{N}_{\varphi ,{te}}}{\sum }_{i=1}^{{N}_{\varphi ,{te}}}{e}^{j{\theta }_{i}}\right|$, where *θ*_*i*_ ~ *Uniform*(0, 2*π*). This expectation value of the residual magnitude of $\tfrac{1}{{N}_{\varphi ,{te}}}{\sum }_{i=1}^{{N}_{\varphi ,{te}}}{e}^{j{\theta }_{i}}$ decreases as *N*_*φ,te*_ increases and would approach zero as *N*_*φ,te*_ → ∞. The numerically simulated output intensity of a diffractive processor design approaches the true time-averaged intensity of the spatially incoherent wave as *N*_*φ,te*_ gets larger, following a similar trend as $\left|\tfrac{1}{{N}_{\varphi ,{te}}}{\sum }_{i=1}^{{N}_{\varphi ,{te}}}{e}^{j{\theta }_{i}}\right|$, reported in Fig. 1d. This comparison also highlights the fact that our choice of using *N*_*φ,te*_ = 20,000 random 2D phase patterns to compute the spatially incoherent output intensity patterns in the blind testing phase is an accurate approximation.

Next, we show in Fig. 2 the scaled intensity linear transformations, $\hat{{\boldsymbol{A}}}$, that were approximated by five of the trained diffractive networks of Fig. 1c. $\hat{{\boldsymbol{A}}}$ is related to the physical transformation ***A***′ by a scalar factor *σ*_*A*_ (see the “Evaluation” subsection in “Materials and methods” section) which compensates for diffraction efficiency-related optical losses. We also show the error matrix with respect to the target ***A***, i.e., ${\boldsymbol{\varepsilon }}{\boldsymbol{=}}{\boldsymbol{|}}{\boldsymbol{A}}-\hat{{\boldsymbol{A}}}{\boldsymbol{|}}$, and report the average of the error matrix elements in the table on the right. Here |∙| denotes the elementwise operation. As *N* increases, the diffractive networks’ resulting matrices resemble the ground truth target better and the approximation error decreases steadily; however, the improvement is more prominent as *N* approaches 2*N*_*i*_ *N*_*o*_ and stagnates beyond *N* ≈ 2*N*_*i*_*N*_*o*_.

To provide visually more noticeable illustrations of the diffractive networks’ all-optical intensity transformations under spatially incoherent illumination, we used structured intensity patterns such as the letters U, C, L, and A as input intensity to the diffractive networks (see Fig. 3). Because of the randomness of the elements of the intensity transformation matrix, the output pixel intensities also appear random (harder to compare visually against the ground truth). However, the reappearance of the letters after a numerical inversion through the multiplication of the scaled output intensity $\hat{{\boldsymbol{o}}}$ by the inverse of the target transformation, ***A***^−1^, would indicate $\hat{{\boldsymbol{A}}}\approx {\boldsymbol{A}}$ and validate the correctness of the diffractive networks’ approximations in a visually noticeable manner (see the “Evaluation” subsection of the “Materials and methods” section for the definition of $\hat{{\boldsymbol{o}}}$). In the case of the diffractive network # 1A (*K* = 5, *N* = 5 × 38^2^ ≈ 0.88 × 2*N*_*i*_*N*_*o*_), the result of such an inversion does not quite reveal any recognizable patterns, indicating the near-failure of the all-optical approximation of this design # 1 A. However, such inversion reveals the recognizable patterns (U, C, L, and A) as *N* approaches 2*N*_*i*_*N*_*o*_ (design # 1B) and becomes identical to the inputs as *N* exceeds 2*N*_*i*_*N*_*o*_ (e.g., design # 1C). These results show that for the *K* = 5 phase-only diffractive networks with a sufficiently large *N* ≥ ~2*N*_*i*_*N*_*o*_, we have $\hat{{\boldsymbol{A}}}\approx {\boldsymbol{A}}$, indicating that these networks could faithfully approximate the target intensity linear transformation under spatially incoherent illumination.

For computational imaging and sensing applications, such as in microscopy, exploring patterns of closely spaced lines and points would be interesting. Motivated by this, we repeated the same procedures outlined in Fig. 3 for various intensity patterns consisting of closely separated line pairs and sets of points, the results of which are summarized in Fig. 4. The same conclusions drawn previously in Fig. 3 hold: for *N* ≥ ~2*N*_*i*_*N*_*o*_ we have $\hat{{\boldsymbol{A}}}\approx {\boldsymbol{A}}$.

We also investigated the dependence of the all-optical approximation of intensity linear transformations on the number of diffractive layers *K*; see Fig. 5. The results of this analysis reveal that even with *N* ≈ 2 × 2*N*_*i*_*N*_*o*_, *K* = 1 and *K* = 2 diffractive designs failed to approximate the target linear transformation despite having a large *N*, whereas the designs with *K* > 2 successfully approximated the target transformation under spatially incoherent illumination. This confirms that the depth of the diffractive network design is a key architectural factor in the computational capacity of diffractive processors to perform arbitrary linear transformations^19,25,40,41^. The diffractive layer phase distributions for different designs with approximately the same *N* ≈ 2 × 2*N*_*i*_*N*_*o*_ diffractive features are shown in Supplementary Fig. S2 for different *K* values. For example, the phase profile of the diffractive layer for *K* = 1 looks significantly different from the layers of the other deeper diffractive networks.

Next, we present the blind testing results of the diffractive processors that were trained using the second design approach (i.e., direct approach), to perform the same arbitrary intensity linear transformation as has been considered so far. In Fig. 6a, the approximation errors of eight different phase-only diffractive processors trained using the direct approach, each with *K* = 5 diffractive layers, are reported as a function of *N*. The mean error was calculated over the same 20,000 test intensity patterns used in Fig. 1c; for each test intensity pattern, the incoherent output intensity ***o***′ was calculated using *N*_*φ,te*_ = 20,000 (same as before). In these alternative diffractive designs, the approximation error of the diffractive processors reaches a minimum level as $\tfrac{N}{2{N}_{i}{N}_{o}}$ approaches 1, and stays at the same level for *N* ≥ 2*N*_*i*_*N*_*o*_ – the same conclusion that we reached for the indirect designs reported earlier. However, compared with the previous designs that used the indirect approach, here, the minimum error level obtained using the direct approach is approximately three times higher. This can be attributed to the use of a relatively small *N*_*φ,tr*_ = 1000 during the training, and these designs can be further improved by increasing *N*_*φ,tr*_ using a longer training effort with more computational resources.

In Fig. 7, we show the scaled linear intensity transformations, $\hat{{\boldsymbol{A}}}$, that were approximated by five of the trained diffractive networks of Fig. 6a. For each case, we also show the error matrix with respect to the target ***A***, i.e., ${\boldsymbol{\varepsilon }}{\boldsymbol{=}}\left|{\boldsymbol{A}}-\hat{{\boldsymbol{A}}}\right|$, and report the average of the error matrix elements in the table on the right. As *N* increases, the mean intensity transformation error decreases, except for design # 2B which we believe is an outlier resulting from poor convergence. The relatively large error of the design # 2B is due to the diffraction efficiency imbalance among the individual input pixels, as evident from the uneven magnitudes across the columns of $\hat{{\boldsymbol{A}}}$. Similarly, the other designs of the direct approach reveal uneven magnitudes across the columns of ***ε***, indicating some diffraction efficiency imbalance among the individual input pixels, albeit not as severe as the design # 2B. Despite such imperfections, these diffractive networks designed using the direct approach effectively learned the target intensity transformation, as evident from Figs. 8 and 9. Figure 8 reveals that, for all the designs, the multiplication of the output intensity patterns $\hat{{\boldsymbol{o}}}$ by the inverse of the target transformation, ***A***^−1^ brings back the patterns U, C, L, A. Although, the reconstruction quality is better for *N* ≈ 2*N*_*i*_*N*_*o*_ and remains similar beyond *N* > 2*N*_*i*_*N*_*o*_, the improvement is not as sharp as it was with the indirect approach (see Fig. 8 vs. Fig. 3 and Fig. 9 vs. Fig. 4). In contrast with the diffractive networks designed using the indirect approach, here in this case, the diffractive networks with *N* < 2*N*_*i*_*N*_*o*_ (e.g., design # 2A) succeeded in approximating the linear transformation to the extent of revealing recognizable patterns after a numerical inverse mapping. These same observations also hold for the intensity patterns that consist of closely spaced lines and points, as shown in Fig. 9.

As another example, we report in Fig. 10 the performance of a diffractive network (*K* = 5, *N* ≈ 2 × 2*N*_*i*_*N*_*o*_) trained using the indirect approach to approximate another arbitrary intensity linear transformation, defined by a non-invertible matrix. The target transformation ***A***, the approximate all-optical transformation $\hat{{\boldsymbol{A}}}$, and the error matrix ${\boldsymbol{\varepsilon }}{\boldsymbol{=}}\left|{\boldsymbol{A}}-\hat{{\boldsymbol{A}}}\right|$ are shown in Fig. 10a, revealing that the diffractive network design performed the target intensity transformation with negligible error. We also show the performance of this diffractive network design on test patterns (U, C, L, and A as well as line pairs and points) in Fig. 10b. The all-optical outputs are identical to the ground truth outputs, further confirming that we have $\hat{{\boldsymbol{A}}}\approx {\boldsymbol{A}}$. Another example of the all-optical approximation of an arbitrary intensity transformation (defined by a random permutation matrix) is also reported in Supplementary Fig. S1.

**Fig. 11:**
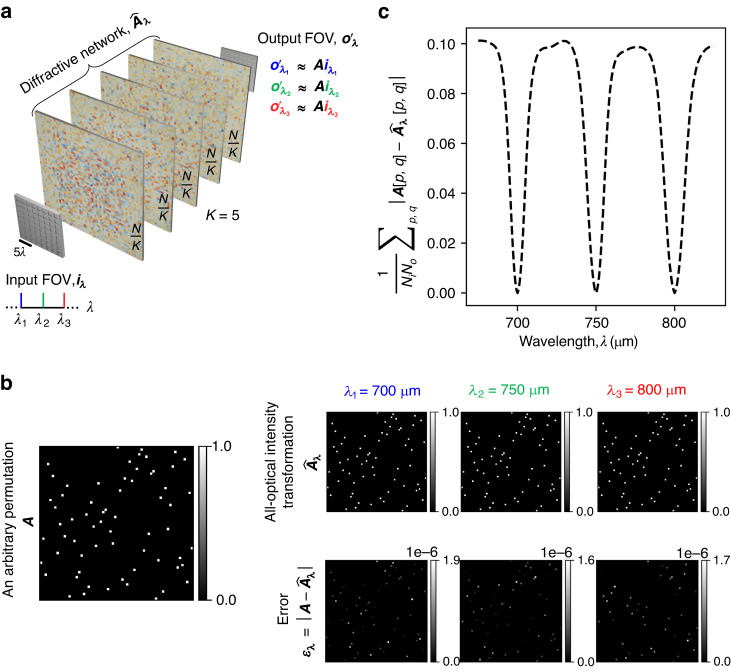
All-optical implementation of the same linear intensity transformation at discrete wavelengths by a diffractive network under spatially incoherent illumination. **a** The spatially incoherent diffractive network simultaneously performs the same intensity linear transformation ***A*** at *N*_*w*_ = 3 discrete wavelengths *λ*_1_, *λ*_2_, *λ*_3_. **b** An arbitrarily chosen target permutation matrix ***A***, together with the all-optical intensity linear transformations (${\hat{{\boldsymbol{A}}}}_{\lambda }$) performed by the trained spatially incoherent diffractive network (*K* = 5, *N* = 5 × 100^2^ ≈ 2.035 × 2*N*_*w*_*N*_*i*_*N*_*o*_) at the three designated wavelengths and the corresponding error matrices ${{\boldsymbol{\varepsilon }}}_{\lambda }=\left|{\boldsymbol{A}}-{\hat{{\boldsymbol{A}}}}_{\lambda }\right|$. Here |**∙**| denotes elementwise operation. **c** Average of the elements of the error matrix ***ε***_*λ*_, as a function of the spatially incoherent illumination wavelength *λ*

**Fig. 12:**
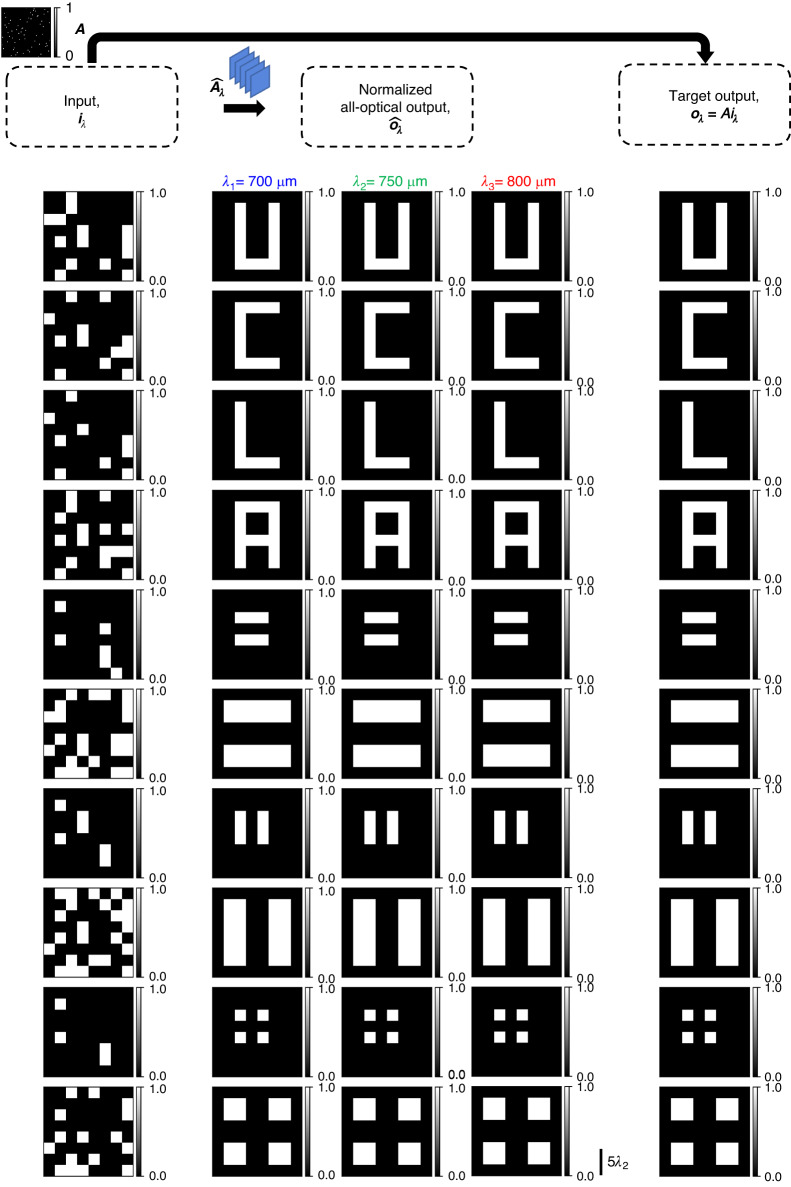
Examples of the same linear transformation on test intensity patterns, performed all-optically by a diffractive network at three separate wavelengths of spatially incoherent illumination. All-optical transformation of test intensity patterns by the trained spatially incoherent diffractive network of Fig. 11, at the three designated wavelengths, together with the corresponding ground truths

**Fig. 13:**
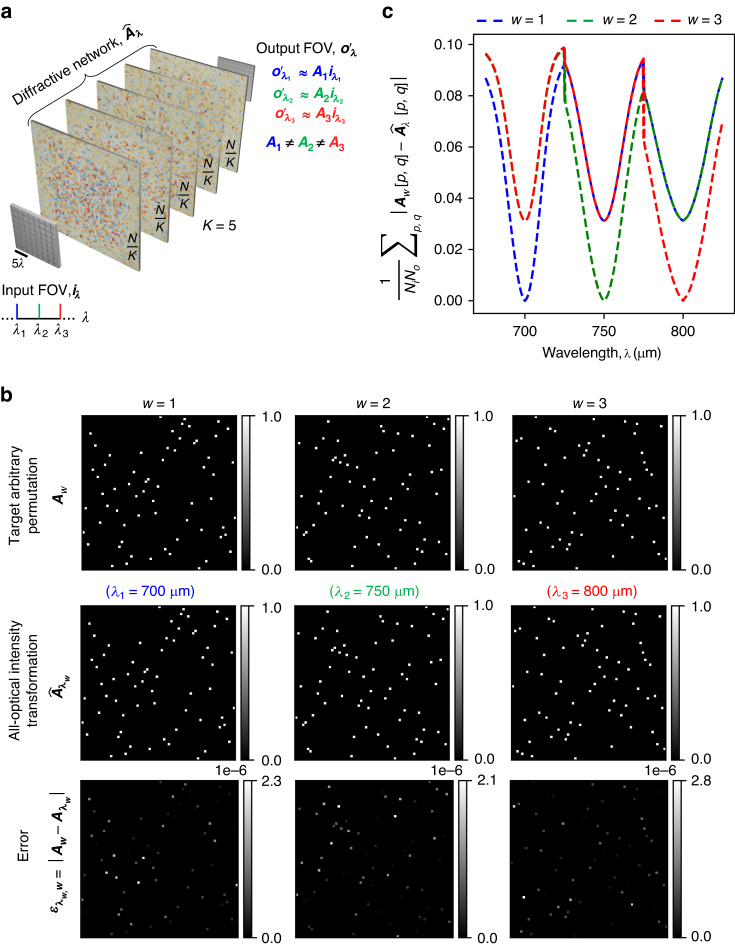
All-optical implementation of multiple unique linear intensity transformations at discrete wavelengths, i.e., wavelength-multiplexing of linear intensity transformations by a diffractive network under spatially incoherent illumination. **a** The spatially incoherent diffractive network simultaneously performs three unique intensity linear transformations ***A***_1_, ***A***_2_, ***A***_3_ at *N*_*w*_ = 3 discrete wavelengths *λ*_1_, *λ*_2_, *λ*_3_, respectively. **b** The target permutation matrices ***A***_1_, ***A***_2_, ***A***_3_, together with the all-optical intensity linear transformations (${\hat{{\boldsymbol{A}}}}_{\lambda }$) performed by the trained spatially incoherent diffractive network (*K* = 5, *N* = 5 × 100^2^ ≈ 2.035 × 2*N*_*w*_*N*_*i*_*N*_*o*_) at the three designated wavelengths and the corresponding error matrices ${{\boldsymbol{\varepsilon }}}_{\lambda ,w}=\left|{{\boldsymbol{A}}}_{w}-{\hat{{\boldsymbol{A}}}}_{\lambda }\right|$. Here |**∙**| denotes elementwise operation. **c** Average of the elements of the error matrix ***ε***_*λ,w*_, as a function of the spatially incoherent illumination wavelength *λ*

**Fig. 14:**
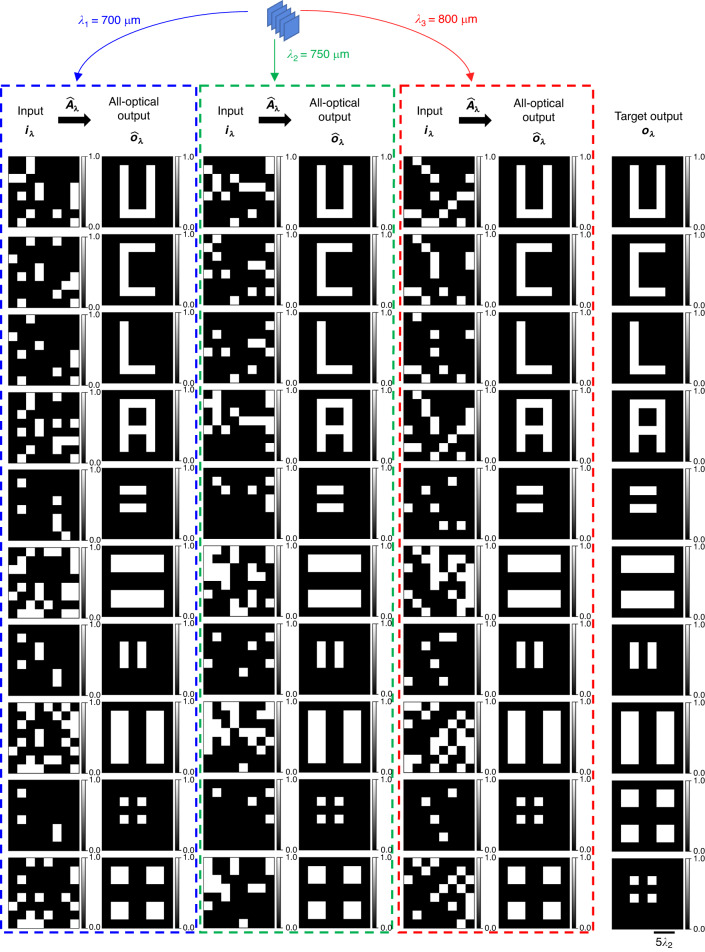
Examples of three unique linear transformations on test intensity patterns, performed all-optically by a diffractive network at three separate wavelengths of spatially incoherent illumination. All-optical transformation of test intensity patterns by the trained spatially incoherent diffractive network of Fig. 13 at the three designated wavelengths, together with the corresponding ground truths

We also demonstrate the capability of spatially incoherent diffractive networks to all-optically perform arbitrary intensity linear transformations at different illumination wavelengths, operating simultaneously. For this purpose, we consider two different cases: (1) the same intensity linear transformation ***A*** is simultaneously performed at *N*_*w*_ = 3 discrete wavelengths *λ*_1_, *λ*_2_, *λ*_3_ (see Figs. 11, 12), and (2) three unique intensity linear transformations ***A***_1_, ***A***_2_, ***A***_3_ are simultaneously performed at *N*_*w*_ = 3 discrete wavelengths *λ*_1_, *λ*_2_, *λ*_3_ (see Figs. 13, 14). For the former, we trained a spatially incoherent diffractive optical network to perform the same arbitrarily chosen permutation matrix ***A***, as shown in Fig. 11b, at *λ*_1_ = 700 μm, *λ*_2_ = 750 μm, *λ*_3_ = 800 μm. The all-optical transformations performed under spatially incoherent light at these three wavelengths, i.e., ${\hat{{\boldsymbol{A}}}}_{{\lambda }_{1}}{\boldsymbol{,}}\,{\hat{{\boldsymbol{A}}}}_{{\lambda }_{2}}$ and ${\hat{{\boldsymbol{A}}}}_{{\lambda }_{3}}$ are also shown in Fig. 11b, together with the corresponding numerical error matrices. We also plot the average of the elements of the error matrices, corresponding to the all-optical transformations ${\hat{{\boldsymbol{A}}}}_{\lambda }$ at different wavelengths in Fig. 11c. These results and analyses show that the spatially incoherent diffractive optical network could simultaneously perform the target permutation with negligible error at these three wavelengths. In Fig. 12, we also depict visual examples of the all-optical permutations performed by the diffractive network. Here we used the inverse-mapped intensities of recognizable test patterns as the input intensities; the diffractive network was successful in all-optically reproducing the test patterns at the output FOV at all three wavelengths with no perceptible error, indicating ${\hat{{\boldsymbol{A}}}}_{{\lambda }_{1}}\,{{\approx}}\,{\hat{{\boldsymbol{A}}}}_{{\lambda }_{2}}\,{{\approx}}\,{\hat{{\boldsymbol{A}}}}_{{\lambda}_{3}}\,{{\approx}}\,{\boldsymbol{A}}$.

For the second case, where we want the spatially incoherent inputs at *λ*_1_, *λ*_2_ and *λ*_3_ to undergo three unique all-optical intensity linear transformations (***A***_1_, ***A***_2_, ***A***_3_, respectively) by the same/common diffractive optical network, we chose three arbitrary permutation matrices such that ∑_*p,q*_***A***_*i*_[*p, q*]***A***_*j*_[*p, q*] = 0 for *i* ≠ *j*. We trained a spatially incoherent diffractive optical network to perform these distinct linear transformations ***A***_1_, ***A***_2_ and ***A***_3_ on the input intensities at *λ*_1_ = 700 μm, *λ*_2_ = 750 μm and *λ*_3_ = 800 μm, respectively. Figure 13b shows the target permutation matrices ***A***_1_, ***A***_2_, ***A***_3_, the resulting all-optical intensity transformations ${\hat{{\boldsymbol{A}}}}_{{\lambda }_{1}}{\boldsymbol{,}}\,{\hat{{\boldsymbol{A}}}}_{{\lambda }_{2}}$, ${\hat{{\boldsymbol{A}}}}_{{\lambda }_{3}}$ performed by the spatially incoherent diffractive network at wavelengths *λ*_1_, *λ*_2_, and *λ*_3_ and the corresponding numerical error matrices. We also plot the average of the elements of the error matrices corresponding to the all-optical transformations ${\hat{{\boldsymbol{A}}}}_{\lambda }$ performed by the diffractive network at different wavelengths *λ*, with respect to the target permutations in Fig. 13c. The results of Figs. 13b, c show that the spatially incoherent diffractive network simultaneously performed the target permutation operations with negligible error at the three designated wavelengths. Apart from the negligible error at the wavelengths designated to the target transforms, the error also has local minima (~0.03125) at the other two wavelengths, as shown in Fig. 13c. This is due to the fact that the all-optical transformations at the other two wavelengths are also permutation operations and the maximum value of the mean absolute error between two unique/non-overlapping permutation matrices of size *N* × *N* is bounded by 2/*N* which is 0.03125 in our case, very well agreeing with the local minima observed in Fig. 13c. In Fig. 14, we also depict some visual examples of the all-optical permutations simultaneously performed by the spatially incoherent diffractive optical network. Similar to Fig. 12, we used the inverse-mapped intensities of recognizable test patterns under ***A***_1_, ***A***_2_ and ***A***_3_ as the input intensities at *λ*_1_ = 700 μm, *λ*_2_ = 750 μm, *λ*_3_ = 800 μm, respectively; the diffractive network all-optically reproduced the test patterns at the output FOV at all three wavelengths with no perceptible error, indicating ${\hat{{\boldsymbol{A}}}}_{{\lambda }_{1}}{\boldsymbol{\approx }}{{\boldsymbol{A}}}_{1}$, ${\hat{{\boldsymbol{A}}}}_{{\lambda }_{2}}{\boldsymbol{\approx }}{{\boldsymbol{A}}}_{2}$, and ${\hat{{\boldsymbol{A}}}}_{{\lambda }_{3}}{\boldsymbol{\approx }}{{\boldsymbol{A}}}_{3}$.

**Fig. 15:**
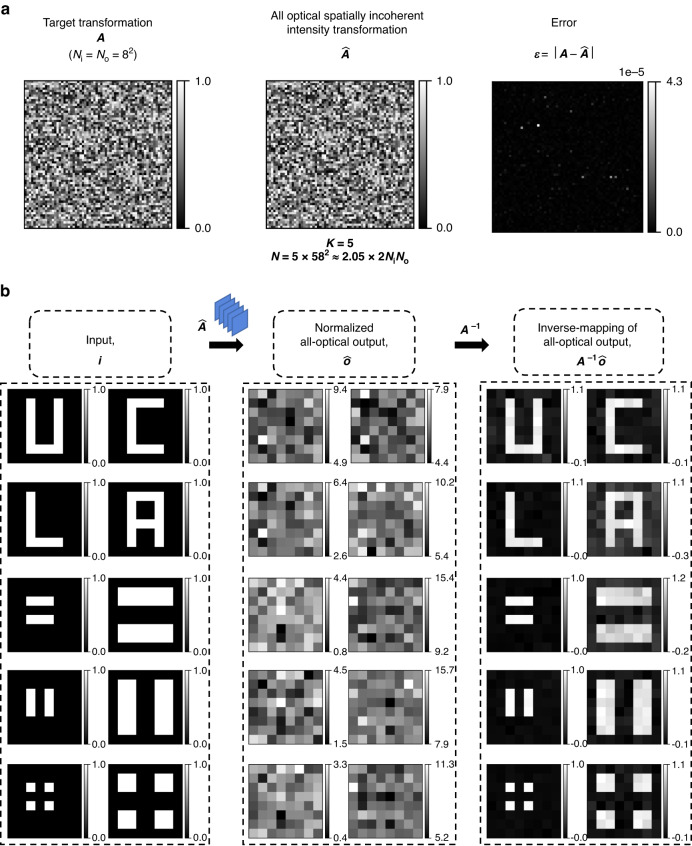
Approximation of an arbitrary 8^2^ × 8^2^ linear transformation (*A*) of intensity, under spatially incoherent illumination, by a diffractive network (*K* = 5, *N* = 5 × 58^2^) trained using the PSF-based data-free design approach. For this design, the size of the intensity pixels is diffraction-limited, i.e., ~λ/2. **a** The target transformation ***A***, the all-optical intensity transformation $\hat{{\boldsymbol{A}}}$ performed by the trained diffractive network and the error matrix ${\boldsymbol{\varepsilon }}=\left|{\boldsymbol{A}}-\hat{{\boldsymbol{A}}}\right|$. Here |**∙**| denotes elementwise operation. **b** All-optical linear transformation of different test intensity patterns by the trained diffractive network, together with the patterns resulting from the numerical inverse mapping of the all-optical outputs through multiplication by ***A***^**−1**^. The all-optical output intensities are simulated using *N*_*φ,te*_ = 100,000

Apart from these indirect and direct design approaches that are both based on ***data-driven supervised learning***, we also used an alternative, third design approach: ***a data-free method based on spatially varying PSFs***. This spatially incoherent diffractive network design approach involves separately propagating each of the *N*_*i*_ input pixels (see Eq. (14) of the “Materials and methods” section) and minimizing the MSE between the all-optical intensity transformation ***A***′ and the target transformation ***A***. Since this approach is based on optimizing the spatially varying PSFs of an incoherent diffractive network, we call it the *PSF-based design approach* that is *data-free*. To showcase the utility of this approach, we trained another spatially incoherent diffractive optical network to perform the same intensity linear transformation ***A*** as in Figs. 1–9, with *N*_*i*_ = *N*_*o*_ = 8 × 8 and a diffraction-limited input/output pixel size of $\sim \frac{\lambda }{2}$. As for the diffractive network architecture, we chose *K* = 5, *N* = 5 × 58^2^ ≈ 2.05 × 2*N*_*i*_*N*_*o*_. Figure 15a shows the target intensity transformation ***A***, the all-optical transformation matrix $\hat{{\boldsymbol{A}}}$ of the trained spatially incoherent diffractive network, and the error matrix ${\boldsymbol{\varepsilon }}{\boldsymbol{=}}\left|{\boldsymbol{A}}-\hat{{\boldsymbol{A}}}\right|$, revealing negligible error in achieving the target linear transformation. We also show the all-optical output intensities for different input test patterns in Fig. 15b, confirming the success of this spatially incoherent diffractive design using the data-free PSF-based approach.

## Discussion

We demonstrated that phase-only diffractive networks under spatially incoherent illumination could perform arbitrary linear transformations of optical intensity with a negligible error if *N* ≥ 2*N*_*i*_*N*_*o*_. The same conclusions would be applicable to complex-valued diffractive networks where the phase and amplitude of each diffractive feature could be independently optimized; in that case, the critical number of complex-valued diffractive features for approximating an arbitrary linear transformation of optical intensity would reduce by half to *N*_*i*_*N*_*o*_ due to the increased degrees of freedom per diffractive layer. Because of the practical advantages of phase-only diffractive networks, without loss of generality, we limited our analyses in this work to phase-only modulation at each diffractive surface.

Our results suggest that the two different data-driven training approaches (indirect vs. direct design) converge differently. If *N* is comparable to or larger than 2*N*_*i*_*N*_*o*_, the indirect approach results in significantly better and faster convergence and accurate approximation $\hat{{\boldsymbol{A}}}\approx {\boldsymbol{A}}$; on the other hand, the direct design approach works better when *N* is considerably less than 2*N*_*i*_*N*_*o*_, even if its approximation error is larger. For example, although the designs # 2A and # 2B have higher errors than the design # 1A, the performances of the former on various test patterns are manifestly better as compared in Figs. 3, 4, 8 and 9. These direct designs can be further improved in their approximation power by increasing *N*_*φ,tr*_ ≫ 1000 through a longer training phase, utilizing more computational resources.

A probable reason for the relatively inferior performance of the indirect design approach for *N* < 2*N*_*i*_*N*_*o*_ is the zero-phase restriction imposed on $\mathop{{\boldsymbol{A}}}\limits^{=}\left[p,q\right]$, i.e., $\mathop{{\boldsymbol{A}}}\limits^{=}\left[p,q\right]=\sqrt{{\boldsymbol{A}}\left[p,q\right]}{{\exp}}\left(j0\right)$. This zero-phase condition might restrict the convergence of the diffractive network design, given limited degrees of freedom, training data and time. Without any such phase restrictions assumed, the direct approach can converge to a relatively better solution for *N* < 2*N*_*i*_*N*_*o*_, satisfying ${\boldsymbol{A}}\left[p,q\right]={\left|\mathop{{\boldsymbol{A}}}\limits^{=}\left[p,q\right]\right|}^{2}$. On the other hand, with *N* ≥ 2*N*_*i*_*N*_*o*_, i.e., with sufficient degrees of freedom available within the network architecture, it becomes easier to meet the additional phase constraint of the indirect design approach, while the direct approach still suffers from training noise arising from limited *N*_*φ,tr*_; this trade-off is at the heart of the relatively inferior performance of the direct approach for *N* ≥ 2*N*_*i*_*N*_*o*_.

**Fig. 16:**
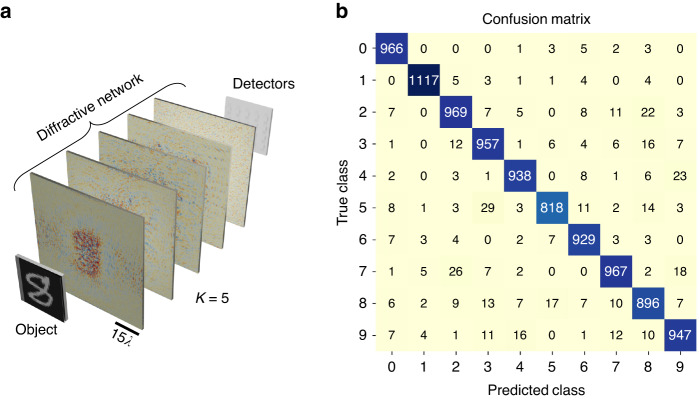
All-optical image classification using a diffractive optical network under spatially incoherent illumination. **a** A *K* = 5 phase-only diffractive network trained to perform all-optical classification of handwritten digits under spatially incoherent illumination. **b** Confusion matrix generated by blind testing of the resulting diffractive network on 10,000 MNIST test images, evaluated using *N*_*φ,te*_ = 20,000. The overall blind testing accuracy of the network on the MNIST test set was 95.04%. For the phase profiles of the trained diffractive network layers, see Supplementary Fig. S4

An important advantage of the direct approach over the indirect one is that the former can be applied even if the only information available to the designer is the sample data representing the target incoherent linear process, without a priori knowledge of the transformation matrix itself. By the same token, the direct approach also lends itself to data-driven optimization of incoherent diffractive processors for all-optical linear approximation of some nonlinear processes. As a consequence of this, data-driven design of incoherent processors for performing other inference tasks such as e.g., all-optical image classification under spatially incoherent illumination, can be accomplished using the direct approach. We demonstrated this important advantage of the direct approach through a practical application involving image classification, i.e., all-optical classification of MNIST handwritten digits^44^ under spatially incoherent illumination. In this scheme, the images are encoded in the intensity of the incoherent illumination, as depicted in Fig. 16a, while at the output plane of the diffractive network, we placed 20 detectors in a differential scheme, i.e., a positive detector and a negative detector for each of the 10 data classes^45^. For training the spatially incoherent diffractive network, we used *N*_*φ,tr*_ = 10 and a batch size of 64. Despite the less accurate forward model with a small *N*_*φ,tr*_, a larger batch size bolstered the training process and facilitated better convergence. Once the model was trained, we used *N*_*φ,te*_ = 20,000 for the blind testing, which resulted in a classification accuracy of 95.04%. The confusion matrix arising from this blind testing of the trained spatially incoherent diffractive network on 10,000 MNIST test images is shown in Fig. 16b. For more details on the training, see the “Materials and methods” section.

Both the indirect and the direct design approaches based on data-driven supervised learning suffer from diffraction efficiency fluctuations across the input pixels to some extent, manifested by the appearance of vertical stripes in some of the all-optical intensity transformations reported in e.g., Figs. 5 and 7. This artifact arises from using a different scaling factor for each example during the training (see Eqs. 11, 13). The artifact is not perceptible for the indirect approach in general, except for the *K* = 2 design shown in Fig. 5 where the artifact is severe. The PSF-based data-free design approach, on the other hand, is free from such artifacts, as shown in Fig. 15, while also being computationally more efficient. For example, the optimization of the spatially incoherent diffractive network reported in Fig. 15 using the data-free PSF-based design approach took less than 4 min. Benefiting from its speed, we also used this PSF-based design approach to tackle a larger problem with *N*_*i*_ = *N*_*o*_ = 16 × 16 as illustrated in Supplementary Fig. S5, for which the optimization took less than 35 min. Despite these advantages, the PSF-based approach, like the indirect design method, cannot be used in the case of an unknown transformation, such as data-driven classification problems, as depicted in Fig. 16.

The failure of shallow diffractive networks to perform an arbitrary intensity transformation (see e.g., *K* = 1 design shown in Fig. 5) indicates that shallow architectures with phase-only diffractive layers are unable to effectively balance the ballistic photons that are transmitted from the sample/input FOV over a low numerical aperture; as a result of this, the lower spatial frequencies of the input intensity patterns dominate the output intensity patterns of a shallow diffractive network, sacrificing the approximation accuracy. Therefore, shallow diffractive network architectures, even with large numbers of trainable diffractive features (*N*), fail to approximate an arbitrary intensity transformation, as shown in Fig. 5. Deeper architectures, on the other hand, utilize their trainable diffractive features more effectively by distributing them across several layers/surfaces, one following another, and mixing the propagating modes of the input FOV over a series of layers that are optimized using deep learning.

As demonstrated in our Results section and Figs. 11–14, spatially incoherent diffractive processor designs can also be extended to operate under broadband illumination light. In fact, multiplexing of *M* > 150 arbitrary complex-valued linear transformations for complex optical fields was shown to be possible under spatially coherent but broadband illumination light^41^. Following a similar multi-wavelength optimization process and the indirect design principles outlined earlier, one can design a diffractive network to simultaneously approximate *M* > 150 arbitrarily-selected linear intensity transformations (${{\boldsymbol{A}}}_{{{\boldsymbol{\lambda }}}_{{\boldsymbol{1}}}}{\boldsymbol{,}}{{\boldsymbol{A}}}_{{{\boldsymbol{\lambda }}}_{{\boldsymbol{2}}}}$,… ${{\boldsymbol{A}}}_{{{\boldsymbol{\lambda }}}_{{\boldsymbol{M}}}}$) under spatially incoherent illumination, where each intensity transformation is assigned to a unique wavelength ***λ***_***i***_ {*i* = 1:*M*}. The success of such a spatially and temporally incoherent diffractive optical network to accurately perform all the target intensity transformations requires an increase in the number of trainable features within the diffractive volume, i.e., *N* ≥ *M* × 2*N*_*i*_*N*_*o*_ would be needed for a phase-only diffractive network. Such diffractive processor designs that work under spatially and temporally incoherent light can be useful for a number of applications, including fluorescence and brightfield microscopy and the processing of natural scenes.

We have limited our analysis to a relatively small problem size, e.g., *N*_*i*_ = *N*_*o*_ = 64 or *N*_*i*_ = *N*_*o*_ = 256 as in Supplementary Fig. S5. Larger problems in terms of *N*_*i*_ and *N*_*o*_ would necessitate diffractive designs with larger *K* and *N*, which in turn would necessitate a longer training phase with a larger training set for converging to a good solution. Even though the PSF-based design approach, as discussed above, could alleviate some of the computational burden, ultimately for megapixel-size input/output problems, distributed training over multiple computers might be necessary for implementing the large optical forward model^46^. As for the physical implementation of a converged diffractive network model, fabrication methods such as lithography and additive manufacturing could be used for creating diffractive layers for high-density incoherent visual computing at the visible and infrared wavelengths^47,48^. While the physical alignment of these diffractive layers might pose some practical challenges, the requirement for precise alignment can be relaxed by training the diffractive processor designs with such fabrication and alignment imperfections added as random physical variables during the training phase; this strategy has been shown to bring resilience against relative misalignments between the fabricated and assembled diffractive layers^49^.

We should also emphasize that the intensity linear transformations performed by diffractive networks under spatially incoherent illumination are not limited to square matrices with *N*_*i*_ = *N*_*o*_. To show this, we trained a diffractive optical network to perform an intensity linear transformation ***A*** with *N*_*i*_ = 64 and *N*_*o*_ = 49, using the indirect design approach. To keep the solution space general, we distributed the pixels on the input and the output FOVs in an irregular manner (arbitrarily selected), completely deviating from the regular 8 × 8 and 7 × 7 grids (see Supplementary Fig. S3a). Supplementary Fig. S3b shows the all-optical intensity transformation $\hat{{\boldsymbol{A}}}$ performed by this trained spatially incoherent diffractive network, together with the target transformation ***A*** and the error matrix ${\boldsymbol{\varepsilon }}{\boldsymbol{=}}\left|{\boldsymbol{A}}-\hat{{\boldsymbol{A}}}\right|$, revealing a negligible transformation error in this case of *N*_*i*_ ≠ *N*_*o*_.

We also note that our framework cannot process negative/complex-valued numbers in its current information encoding implementation since it uses optical intensity to represent information. However, it can be extended to implement complex-valued transformations by mapping and encoding the complex numbers, e.g., real and imaginary parts, as well as negative numbers to be represented by optical intensity.

## Materials and methods

### Model for the propagation of spatially coherent light through a diffractive optical network

Propagation of spatially coherent complex optical fields through a diffractive processor ${\mathfrak{D}}\left\{\cdot \right\}$ constitutes successive amplitude and/or phase modulation by diffractive surfaces, each followed by coherent propagation through the free space separating consecutive diffractive surfaces. The diffractive features of a surface locally modulate the incident optical field *u*(*x, y*). For this paper, the trainable diffractive features are *phase-only*, i.e., only the phase, but not the amplitude, of the incident field is modulated by the diffractive surface. In other words, the field immediately after the surface would be *u*(*x, y*) exp(*jϕ*_*M*_(*x, y*)) where the local phase change *ϕ*_*M*_(*x, y*) induced by the surface is related to its height *h*(*x, y*) as ${\phi }_{M}=\frac{2\pi }{\lambda }\left(n-1\right)h$. Here *n* is the refractive index of the diffractive surface material.

Free-space propagation of an optical field between consecutive diffractive surfaces was modeled using the angular spectrum method^1^, according to which the propagation of an optical field *u*(*x, y*) by distance *d* can be computed as follows:

6 $$u\left(x,y{\rm{;}}\,z={z}_{0}+d\right)={{\mathcal{F}}}^{-1}\left\{{\mathcal{F}}\left\{u\left(x,y{\rm{;}}\,z={z}_{0}\right)\right\}\times H\left({f}_{x},{f}_{y}{\rm{;}}\,d\right)\right\}$$

where ${{\mathcal{F}}}$ $({{\mathcal{F}}}^{-1})$ is the two-dimensional Fourier (Inverse Fourier) transform and *H*(*f*_*x*_, *f*_*y*_; *d*) is the free-space transfer function for an axial propagation distance *d*:

7 $$H\left({f}_{x},{f}_{y}{{;}}d\right)=\left\{\begin{array}{c}{{\exp}}\left(j\frac{2\pi }{\lambda }d\sqrt{1-{\left({\lambda f}_{x}\right)}^{2}-{\left({\lambda f}_{y}\right)}^{2}}\right),\,{f}_{x}^{2}+{f}_{y}^{2} \,< 1/{\lambda }^{2}\\ \qquad\qquad\qquad\qquad\qquad\quad\, 0,\quad{\rm{otherwise}}\end{array}\right.$$

where *λ* is the wavelength of light.

### Model for the propagation of spatially incoherent light through a diffractive optical network

With spatially incoherent light, the (average) output optical intensity *O*(*x*, *y*) of a diffractive network, for a given input intensity *I*(*x*, *y*), can be written as

8 $$O\left(x,y\right)=\left\langle {\left|{\mathfrak{D}}\left\{\sqrt{I\left(x,y\right)}{{\exp}}\left(j\varphi \left(x,y\right)\right)\right\}\right|}^{2}\right\rangle =\mathop{{\rm{lim}}}\limits_{{N}_{\varphi }\to {{\infty }}}\frac{1}{{N}_{\varphi }}\mathop{\sum }\limits_{r=1}^{{N}_{\varphi }}{\left|{\mathfrak{D}}\left\{\sqrt{I\left(x,y\right)}{{\exp}}\left(j{\varphi }_{r}\left(x,y\right)\right)\right\}\right|}^{2}$$

where ${\mathfrak{D}}\left\{\cdot \right\}$ denotes the coherent propagation of the optical field through the diffractive processor as described in the preceding subsection, and ${\langle}\cdot{\rangle}$ denotes the statistical average, over all the realizations of the spatially independent random process *φ*(*x, y*) representing the 2D phase of the input optical field, i.e., *φ*(*mδ, nδ*) ~ *Uniform*(0, 2*π*) for all *m*, *n*^43^.

As for the spatially incoherent propagation of average intensity, it is only possible to approximate the true average (Eq. 8) by averaging over a finite number *N*_*φ*_ of samples of *φ*(*x*, *y*), i.e.,

9 $$O\left(x,y\right)\approx \frac{1}{{N}_{\varphi }}\mathop{\sum }\limits_{r=1}^{{N}_{\varphi }}{\left|{\mathfrak{D}}\left\{\sqrt{I\left(x,y\right)}{{\exp}}\left(j{\varphi }_{r}\left(x,y\right)\right)\right\}\right|}^{2}$$

In the training phase of the direct training approach, incoherent propagation of intensities through the diffractive processors was simulated with *N*_*φ,tr*_ = 1000. However, in the blind testing phase we used *N*_*φ,te*_ = 20,000 while evaluating the diffractive processors once they were trained, irrespective of whether the indirect or the direct approach of training was used.

In our numerical simulations, the fields/intensities were discretized using *δ* ≈ 0.53*λ* along both *x* and *y*, e.g., $u\left(m,n\right)\triangleq u\left(m\delta ,n\delta \right)$ and sufficiently zero-padded before evaluating the Fourier transform, as in Eq. (6), using Fast Fourier Transform (FFT) algorithm. In particular, the fields were zero-padded such that the simulation window size after padding was four-times the size of the largest aperture, which in our case is the diffractive layer width. Such sampling ensured that the propagation distance *d* was smaller than the largest propagation distance for which the angular spectrum method is valid, satisfying the sampling requirement for accurate diffraction calculations^50^.

The angular spectrum method, which we used to model the light propagation between diffractive layers, is a Fourier transform-based fast implementation of the Rayleigh-Sommerfeld diffraction integral^1^. By using the Rayleigh-Sommerfeld model of diffraction, we implicitly assumed that the light traveling through these layers can be represented as a scalar field. While the accurate modeling and computation of diffracted light fields from structures with deeply subwavelength features require the use of vector diffraction theory, we made certain assumptions that allowed us to utilize the scalar field approximation. Firstly, we assumed that the diffractive layers are axially separated from each other by more than a wavelength (*d* ≫ *λ*), prohibiting the coupling of evanescent fields from one layer to the next. Secondly, we considered the smallest feature size on a diffractive layer to be approximately half a wavelength. These assumptions permitted us to approximate the spatial information flow within a diffractive optical network using scalar optical fields. In fact, the same scalar field approximation is ubiquitously employed in simulating and modeling diffraction-limited microscopy, holographic imaging and display systems. Various experimental demonstrations of 3D-fabricated diffractive optical networks were reported in the literature, which employed the same scalar field theory^25,30,33,38,41^ providing an excellent match between the numerical and experimental results. These demonstrations further confirm the validity of the scalar field approximation to represent the behavior of light propagation within diffractive optical networks that only process propagating, i.e., traveling waves in space.

### Diffractive network architecture

The heights $h\left(m,n\right)\triangleq h\left(m\delta ,n\delta \right)$ of the *N* diffractive features distributed over *K* surfaces were optimized for designing the diffractive processors to perform the desired transformation. To keep the connectivity between successive diffractive layers^25^ the same across the trained diffractive networks with different *N*, the layer-to-layer separation was set as $d=\tfrac{W\delta }{\lambda }$, where $W=\sqrt{\tfrac{N}{K}}\delta$ is the width of each diffractive layer. The distances between the input FOV and layer-1 and between layer-*K* and the output FOV were also set as *d*. The pixel size on both the input and the output FOVs was ~2.13*λ* × 2.13*λ*, i.e., 4*δ* × 4*δ*.

### Linear transformation matrix

In this paper, the input and the output of the diffractive networks have dimensions of *N*_*i*_ = *N*_*o*_ = 8 × 8, i.e., $I,{O}\in {{\mathbb{R}}}_{+}^{8\times 8}$ and ${\boldsymbol{i}},\,{\boldsymbol{o}}\in {{\mathbb{R}}}_{+}^{64}$. To clarify, ***i*** and ***o*** are one-dimensional (column) vectors obtained by rearranging the intensity values *I*(*m*, *n*) and *O*(*m*, *n*) of the input and the output pixels arranged in a two-dimensional 8 × 8 square grid. Accordingly, the target transformation matrix ***A*** has a size of *N*_*o*_ × *N*_*i*_ = 64 × 64, i.e., ${\boldsymbol{A}}\in {{\mathbb{R}}}_{+}^{64\times 64}$.

### The indirect approach of training

*Dataset preparation*: In the indirect approach, instead of training the diffractive networks to perform the linear transformation ***A*** between the input and the output intensities, we trained them to perform the complex-valued linear transformation $\overline{\overline{{\boldsymbol{A}}}}$ between the input and the output fields such that $\mathop{{\boldsymbol{A}}}\limits^{=}\left[p,q\right]=\sqrt{{\boldsymbol{A}}\left[p,q\right]}{{\exp}}\left(j0\right)$. To prepare the dataset for such training, we first generated input field vectors $\widetilde{{\boldsymbol{i}}}$ with complex-valued elements, where the amplitudes were sampled independently from each other from the uniform distribution *Uniform*(0,1) and the phases from the distribution *Uniform*(0, 2*π*). Then we used the relationship $\widetilde{{\boldsymbol{o}}}{\boldsymbol{=}}\mathop{{\boldsymbol{A}}}\limits^{=}\widetilde{{\boldsymbol{i}}}$ to generate the target ground truths for the corresponding output field vectors. We generated 160,000 such pairs and split them into training and validation sets with a ratio of 15:1.

*Loss function*: We used MSE between the target output field and the all-optical output field of the diffractive processor as the loss function to minimize for optimizing the diffractive surface thicknesses, i.e., the loss function was defined as:

10 $${{\mathcal{L}}}_{{indirect}}=\frac{1}{{N}_{o}}\sum\limits_{l=1}^{{N}_{o}}|\mathop{\sigma }\limits^{=}\widetilde{{\boldsymbol{o}}}\left[l\right]-{\mathop{\sigma }\limits^{=}}^{{\prime} }{\widetilde{{\boldsymbol{o}}}}^{{\boldsymbol{{\prime} }}}[l]|^{2}$$

where ${\widetilde{{\boldsymbol{o}}}}^{{\boldsymbol{{\prime} }}}$ is the diffractive network output field evaluated by coherent propagation of the input field through the diffractive network, $\mathop{\sigma }\limits^{=}$ and ${\mathop{\sigma }\limits^{=}}^{{\boldsymbol{{\prime} }}}$ are normalization factors defined as^19^:

11 $$\mathop{\sigma }\limits^{=}={\left(\mathop{\sum }\limits_{l=1}^{{N}_{o}}{{|}\widetilde{{\boldsymbol{o}}}\left[l\right]{|}}^{2}\right)}^{-\frac{1}{2}},\,{\mathop{\sigma }\limits^{=}}^{{\prime} }=\frac{\sum \nolimits_{l=1}^{{N}_{o}}\mathop{\sigma }\limits^{=}\widetilde{{\boldsymbol{o}}}\left[l\right]{{\widetilde{{\boldsymbol{o}}}}^{{{{\prime} }}}}{}^{{\boldsymbol{* }}}\left[l\right]}{\sum \nolimits_{l=1}^{{N}_{o}}{{{|}}{\widetilde{{\boldsymbol{o}}}}^{{{{\prime} }}}\left[l\right]{{|}}}^{2}}$$

### The direct approach of training

*Dataset preparation:* To prepare the dataset for training diffractive processors for a given transformation ***A*** with the direct approach, we first generated input intensity vectors ***i*** with elements (pixel values) sampled independently from each other from the uniform distribution *Uniform*(0,1). Corresponding ground truths for the output intensity vectors were calculated as ***o*** = ***Ai***. In total, we generated 160,000 pairs of intensity vectors and split them into training and validation sets with a ratio of 15:1.

*Loss function:* The loss function was defined as follows:

12 $${{\mathcal{L}}}_{{direct}}=\frac{1}{{N}_{o}}\mathop{\sum }\limits_{l=1}^{{N}_{o}}{\left(\sigma {\boldsymbol{o}}\left[l\right]-{\sigma }^{{{{\prime} }}}{{\boldsymbol{o}}}^{{{{\prime} }}}\left[l\right]\right)}^{2}$$

where ***o***′ is the diffractive network output intensity evaluated by simulating incoherent propagation of the input intensity through the diffractive network, *σ* and *σ*′ are normalization factors defined as:

13 $$\sigma ={\left(\mathop{\sum }\limits_{l=1}^{{N}_{o}}{\left({\boldsymbol{o}}\left[l\right]\right)}^{2}\right)}^{-\frac{1}{2}},\,{\sigma }^{{{{\prime} }}}=\frac{\sum \nolimits_{l=1}^{{N}_{o}}\sigma {\boldsymbol{o}}\left[l\right]{{\boldsymbol{o}}}^{{{{\prime} }}}\left[l\right]}{\sum \nolimits_{l=1}^{{N}_{o}}{\left({{\boldsymbol{o}}}^{{{{\prime} }}}\left[l\right]\right)}^{2}}$$

Note that during the training of the diffractive networks, the diffraction efficiency was not forced to be uniform across training examples and as a result the scaling factor for the output intensity $\tfrac{\sigma {\boldsymbol{{\prime} }}}{\sigma }$ varies for different inputs. Therefore, the diffractive networks trained using the direct approach exhibit unbalanced diffraction efficiency across the input pixels, as indicated by the uneven brightness across the columns (see e.g., Fig. 7); however, with increasing *N*, such unbalance becomes less severe. Although the same is true for the indirect approach, this unbalance in diffraction efficiency is less severe, except for the *K* = 2 design shown in Fig. 5.

### The PSF-based data-free design approach

The PSF-based optimization was performed by minimizing the MSE loss between the all-optical intensity transformation ***A***′ performed by the spatially incoherent diffractive network and the target transformation ***A***. To evaluate ***A***′, we used *N*_*i*_ intensity vectors ${\left\{{{\boldsymbol{i}}}_{t}\right\}}_{t=1}^{{N}_{i}}$ where ***i***_*t*_[*l*] = 1 if *l* = *t* and 0 otherwise. In other words, ${\left\{{{\boldsymbol{i}}}_{t}\right\}}_{t=1}^{{N}_{i}}$ represent the unit impulse functions located at different input pixels. We simulated the all-optical output intensity vectors ${\left\{{{\boldsymbol{o}}}_{t}^{{\boldsymbol{{\prime} }}}\right\}}_{t=1}^{{N}_{i}}$ corresponding to these input intensity vectors, and stacked them column by column, i.e.,

14 $${{\boldsymbol{A}}}^{{\boldsymbol{{\prime} }}}{\boldsymbol{=}}\left[{{\boldsymbol{o}}}_{1}^{{\boldsymbol{{\prime} }}}{\rm{|}}{{\boldsymbol{o}}}_{2}^{{\boldsymbol{{\prime} }}}{\rm{|}}\cdots {\rm{|}}{{\boldsymbol{o}}}_{{N}_{i}}^{{\boldsymbol{{\prime} }}}\right]$$

The loss function was defined as:

15 $${{\mathcal{L}}}_{{PSF}}=\frac{1}{{{N}_{i}N}_{o}}\sum\limits_{q=1}^{{N}_{i}}\mathop{\sum }\limits_{p=1}^{{N}_{o}}{\left({\boldsymbol{A}}\left[p,q\right]-{\sigma }_{A}^{{{{\prime} }}}{{\boldsymbol{A}}}^{{{{\prime} }}}\left[p,q\right]\right)}^{2}$$

where

16 $${\sigma }_{A}^{{{{\prime} }}}=\frac{\sum\nolimits _{q=1}^{{N}_{i}}\sum \nolimits_{p=1}^{{N}_{o}}{\boldsymbol{A}}\left[p,q\right]{{\boldsymbol{A}}}^{{{{\prime} }}}\left[p,q\right]}{\sum \nolimits_{q=1}^{{N}_{i}}\sum \nolimits_{p=1}^{{N}_{o}}{\left({{\boldsymbol{A}}}^{{{{\prime} }}}\left[p,q\right]\right)}^{2}}$$

### Other training details

The height *h* of the diffractive features at each layer was confined between zero and a maximum value *h*_*max*_ by using a latent variable *h*_*latent*_:

17 $$h=\frac{{h}_{max}}{2}\times \left[\sin \left({h}_{{latent}}\right)+1\right]$$

We chose ${h}_{max}\approx \frac{\lambda }{n-1}$ so that the corresponding phase modulation depth is 2*π*. The latent variables were initialized randomly from the standard normal distribution *N* (0, 1).

In the indirect and the direct design approaches that are data-driven, the diffractive layers were optimized using the AdamW optimizer^51^ for 50 epochs with a minibatch size of 8 and an initial learning rate of 10^−3^. The learning rate was decayed by a factor of 0.7 every five epochs. We evaluated the mean loss of the trained model on the validation set after the completion of each epoch and selected the trained model state at the end of the epoch corresponding to the lowest validation loss. These details were the same for both the indirect and the direct training approaches. For the PSF-based data-free design approach, the diffractive layers were optimized using the AdamW optimizer for 12,000 iteration steps with an initial learning rate of 10^−1^. The learning rate was decayed by a factor of 0.5 if the loss did not decrease for 20 iteration steps, using the PyTorch built-in class: *torch.optim.lr_scheduler*.*ReduceLROnPlateau*.

The diffractive processor models were implemented and trained using PyTorch (v1.10)^52^ with Compute Unified Device Architecture (CUDA) version 11.3.1. Training and testing were done on GeForce RTX 3090 graphics processing units (GPU) in workstations with 256GB of random-access memory (RAM) and Intel Core i9 central processing unit (CPU). The training time of the models varied with the training approach as well as the size of the models in terms of *K* and *N*. For example, the indirect training of *K* = 5, *N* = 5 × 52^2^ diffractive network model took less than 2 h, whereas with the direct approach, the training time for the *K* = 5, *N* = 5 × 52^2^ model with *N*_*φ,tr*_ = 1000 was around 10 days. With the PSF-based data-free design approach, all the 12,000 update steps took in total <4 min (Fig. 15).

### Evaluation

The evaluation procedure was the same across all the trained diffractive networks irrespective of whether the direct approach or the indirect approach was used to train them. To evaluate the trained diffractive networks, we generated a test set comprising 20,000 pairs of input and target intensity vectors ***o*** = ***Ai***. Note that these 20,000 test examples were generated using a different random seed from the ones used to generate the training and the validation sets to ensure they were not represented during the training. For a given ***i***, the corresponding input intensity pattern was incoherently propagated through the trained diffractive network (as in Eq. 9) using *N*_*φ,te*_ = 20,000 to compute the output intensity ***o***′. The mean of the error between ***o***′ and ***o*** (Eq. 12) over the 20,000 test examples was used to quantify the output error of the diffractive network for comparing different designs, as in Figs. (1) and (6). For comparison between the ground truth and the all-optical output intensities, e.g., in Figs. 3, 4, 8, 9, 10, we defined the scaled all-optical output intensity vector $\hat{{\boldsymbol{o}}}=\frac{\sigma {\prime} }{\sigma }{\boldsymbol{o}}{\prime}$, where the definitions of *σ* and *σ*′ are as described in Eq. (13).

The intensity transformation ***A***′ performed by the spatially incoherent diffractive network at the end of its training was evaluated following Eq. (14). However, considering the diffraction-efficiency-associated scaling mismatch between ***A***′ and the target transformation ***A***, we defined a scaled diffractive network intensity transformation $\hat{{\boldsymbol{A}}}={\sigma }_{A}{\boldsymbol{A}}$, where:

18 $${\sigma }_{A}=\sqrt{\frac{\sum \nolimits_{q=1}^{{N}_{i}}\sum \nolimits_{p=1}^{{N}_{o}}{({\boldsymbol{A}}[p,q])}^{2}}{\sum \nolimits_{q=1}^{{N}_{i}}\sum \nolimits_{p=1}^{{N}_{o}}{({\boldsymbol{A}}{{^{\prime} }}[p,q])}^{2}}}$$

This definition of *σ*_*A*_ makes the 2-norms of ***A*** and $\hat{{\boldsymbol{A}}}$ equal.

### Diffractive optical network training for multi-wavelength spatially incoherent illumination

We used the indirect design approach for training diffractive networks to perform wavelength-multiplexed intensity linear transformations under spatially incoherent illumination. The loss function ${\mathcal L}$ was defined as:

19 $${\mathcal L} =\frac{1}{{N}_{w}}\mathop{\sum }\limits_{w=1}^{{N}_{w}}{\alpha }_{w}{ {\mathcal L} }_{indirect,w}$$

Here *N*_*w*_ = 3 is the number of wavelength channels used and ${ {\mathcal L} }_{indirect,w}$ is the MSE loss as defined in Eq. (10), computed using the target output field ${\tilde{{\boldsymbol{o}}}}_{w}={\overline{\overline{{\boldsymbol{A}}}}}_{w}{\tilde{{\boldsymbol{i}}}}_{w}$ and the all-optical output field ${\tilde{{\boldsymbol{o}}}}_{w}^{\text{'}}$ at wavelength *λ*_*w*_; the associated normalization factors ${\overline{\overline{\sigma }}}_{w}$ and ${\overline{\overline{\sigma }}}_{w}^{{\prime} }$ were defined similarly as in Eq. (11). To clarify, ${\overline{\overline{{\boldsymbol{A}}}}}_{w}[p,q]=\sqrt{{{\boldsymbol{A}}}_{{\boldsymbol{w}}}[p,q]}\exp (j0)$ where ***A***_***w***_ is the target intensity linear transformation at the wavelength *λ*_*w*_.

Adaptive spectral weight coefficients *α*_*w*_ were used to balance the performance across the wavelength channels^41^. The initial values of *α*_*w*_ were set as 1 for all *w*, and updated after each training step according to the following rule:

20 $${\alpha }_{w}\leftarrow \,\max (0.1\times ({ {\mathcal L} }_{indirect,w}-{ {\mathcal L} }_{indirect,0})+{\alpha }_{w},0)$$

The refractive indices *n*_*w*_ of the diffractive layer material at the terahertz wavelengths *λ*_*1*_ = 700 μm, *λ*_*2*_ = 750 μm and *λ*_*3*_ = 800 μm were assumed to be 1.7258, 1.7224, and 1.7194, respectively. The maximum layer height hyperparameter *h*_*max*_ was set as 1.2 mm and the diffractive layer feature size was assumed to be 0.4 mm.

### Spatially incoherent diffractive network training for image classification

For the all-optical image classification task reported in Fig. 16, the numerical simulations were performed in the visible range, where we used *λ* = 490 nm and a diffractive feature size of 200 nm to emulate incoherent visible light in natural scenes. The MNIST handwritten digit images were normalized to [0–1] and upsampled to 80 × 80 pixels. The diffractive network comprised five phase-only diffractive layers, each containing 160 × 160 diffractive features. At the output plane of the diffractive network, 20 detectors were arranged in a differential scheme, i.e., a “positive” detector and a “negative” detector were used for each of the 10 data classes^45^. The computational window size was set to 512 × 512.

For image classification, the differential class scores were computed as:

21 $${s}_{c}=\frac{{I}_{c,+}-{I}_{c,-}}{{I}_{c,+}+{I}_{c,-}}$$

Here, *I*_*c*,+_ and *I*_*c*,−_ are the integrated intensity over the positive and negative detectors, corresponding to data class *c*. The class corresponding to the maximum *s*_*c*_ was selected as the inferred object class.

The spatially incoherent diffractive network classifier of Fig. (16) was trained using the cross-entropy loss, i.e.,

22 $${\mathcal L} =-\mathop{\sum }\limits_{c=0}^{9}{\delta }_{ck}\,\log \frac{\exp (\beta {s}_{c})}{\sum \nolimits_{i=0}^{9}\exp (\beta {s}_{i})}$$

Here *k* is the ground truth label and *δ*_*ck*_ is the Kronecker delta function. *β* = 10 is a training hyperparameter. In the training, we used *N*_*φ,tr*_ = 10 with a batch size of 64. The diffractive network was trained for 500 epochs with AdamW optimizer initiated with a learning rate of 10^−4^. The final model was selected based on the validation accuracy with *N*_*φ,tr*_ = 10. After the training, the selected model was blindly tested using *N*_*φ,te*_ = 20,000, which resulted in a classification test accuracy of 95.04% (see Fig. 16).

### Supplementary information

Supplementary Information

## References

[1] Goodman, J. W. *Introduction to Fourier Optics*. 3rd ed. (Greenwoood: Roberts & Company Publishers, 2005).

[2] Athale R, Psaltis D (2016). Optical computing: past and future. Opt. Photonics News.

[3] Solli DR, Jalali B (2015). Analog optical computing. Nat. Photonics.

[4] Mengu D (2022). At the intersection of optics and deep learning: statistical inference, computing, and inverse design. Adv. Opt. Photonics.

[5] Lugt AV (1964). Signal detection by complex spatial filtering. IEEE Trans. Inf. Theory.

[6] Heinz RA, Artman JO, Lee SH (1970). Matrix multiplication by optical methods. Appl. Opt..

[7] Goodman JW, Woody LM (1977). Method for performing complex-valued linear operations on complex-valued data using incoherent light. Appl. Opt..

[8] Tamura PN, Wyant JC (1979). Two-dimensional matrix multiplication using coherent optical techniques. Opt. Eng..

[9] Spall J (2020). Fully reconfigurable coherent optical vector–matrix multiplication. Opt. Lett..

[10] Goodman JW, Dias AR, Woody LM (1978). Fully parallel, high-speed incoherent optical method for performing discrete Fourier transforms. Opt. Lett..

[11] Stark, H. *Application of Optical Fourier Transforms*. (Elsevier Science, Amsterdam, Netherlands, 2012).

[12] Farhat NH (1985). Optical implementation of the hopfield model. Appl. Opt..

[13] Zuo Y (2019). All-optical neural network with nonlinear activation functions. Optica.

[14] Hotate K, Okugawa T (1994). Optical information processing by synthesis of the coherence function. J. Lightwave Technol..

[15] Silva A (2014). Performing mathematical operations with metamaterials. Science.

[16] Kwon H (2018). Nonlocal metasurfaces for optical signal processing. Phys. Rev. Lett..

[17] Zangeneh-Nejad F (2021). Analogue computing with metamaterials. Nat. Rev. Mater..

[18] Yu N, Capasso F (2014). Flat optics with designer metasurfaces. Nat. Mater..

[19] Kulce O (2021). All-optical synthesis of an arbitrary linear transformation using diffractive surfaces. Light Sci. Appl..

[20] Banerji S (2020). Extreme-depth-of-focus imaging with a flat lens. Optica.

[21] Xu N, Liu G, Tan Q (2020). Adjustable super-resolution microscopy with diffractive spot array illumination. Appl. Phys. Lett..

[22] Baek, S. H. et al. Single-shot hyperspectral-depth imaging with learned diffractive optics. In *Proc. IEEE/CVF International Conference on Computer Vision* 2651-2660 (IEEE, Montreal, 2021).

[23] Xu N, Liu G, Tan Q (2022). High-fidelity far-field microscopy at λ/8 resolution. Laser Photonics Rev..

[24] Xu, N. et al. Mechanical-scan-free and multi-color super-resolution imaging with diffractive spot array illumination. Print at 10.48550/arXiv.2303.06988 (2023). 10.1038/s41467-024-48482-z PMC11099116 38755150

[25] Lin X (2018). All-optical machine learning using diffractive deep neural networks. Science.

[26] Mengu D (2019). Analysis of diffractive optical neural networks and their integration with electronic neural networks. IEEE J. Sel. Top. Quantum Electron..

[27] LeCun Y, Bengio Y, Hinton G (2015). Deep learning. Nature.

[28] Rahman MSS (2021). Ensemble learning of diffractive optical networks. Light Sci. Appl..

[29] Li J (2021). Spectrally encoded single-pixel machine vision using diffractive networks. Sci. Adv..

[30] Luo Y (2022). Computational imaging without a computer: seeing through random diffusers at the speed of light. eLight.

[31] Rahman MSS, Ozcan A (2021). Computer-free, all-optical reconstruction of holograms using diffractive networks. ACS Photonics.

[32] Mengu D, Ozcan A (2022). All-optical phase recovery: diffractive computing for quantitative phase imaging. Adv. Opt. Mater..

[33] Bai B (2022). To image, or not to image: class-specific diffractive cameras with all-optical erasure of undesired objects. eLight.

[34] Rahman MSS, Ozcan A (2023). Time-lapse image classification using a diffractive neural network. Adv. Intell. Syst..

[35] Bai, B. et al. Data-class-specific all-optical transformations and encryption. *Adv. Mater.*10.1002/adma.202212091 (2023). 10.1002/adma.202212091 37186024

[36] Goi E, Schoenhardt S, Gu M (2022). Direct retrieval of zernike-based pupil functions using integrated diffractive deep neural networks. Nat. Commun..

[37] Luo X (2022). Metasurface-enabled on-chip multiplexed diffractive neural networks in the visible. Light Sci. Appl..

[38] Luo Y (2019). Design of task-specific optical systems using broadband diffractive neural networks. Light Sci. Appl..

[39] Veli M (2021). Terahertz pulse shaping using diffractive surfaces. Nat. Commun..

[40] Li J (2022). Polarization multiplexed diffractive computing: all-optical implementation of a group of linear transformations through a polarization-encoded diffractive network. Light Sci. Appl..

[41] Li J (2023). Massively parallel universal linear transformations using a wavelength-multiplexed diffractive optical network. Adv. Photonics.

[42] Shannon CE (1949). Communication in the presence of noise. Proc. IRE.

[43] Saleh, B. E. A. & Teich, M. C. *Fundamentals of Photonics*. (Hoboken: Wiley, 2007).

[44] Lecun Y (1998). Gradient-based learning applied to document recognition. Proc. IEEE.

[45] Li J (2019). Class-specific differential detection in diffractive optical neural networks improves inference accuracy. Adv. Photonics.

[46] Chahal KS (2020). A Hitchhiker’s guide on distributed training of deep neural networks. J. Parallel Distrib. Comput..

[47] Saha SK (2019). Scalable submicrometer additive manufacturing. Science.

[48] Beaman JJ (2020). Additive manufacturing review: early past to current practice. J. Manuf. Sci. Eng..

[49] Mengu D (2020). Misalignment resilient diffractive optical networks. Nanophotonics.

[50] Kozacki T, Falaggis K (2015). Angular spectrum-based wave-propagation method with compact space bandwidth for large propagation distances. Opt. Lett..

[51] Loshchilov, I. & Hutter, F. Decoupled weight decay regularization. In *Proc. 7th International Conference on Learning Representations* (New Orleans, ICLR, 2019).

[52] Paszke, A. et al. PyTorch: an imperative style, high-performance deep learning library. In *Proc. 33rd International Conference on Neural Information Processing Systems* 721 (Vancouver, Curran Associates Inc., 2019).

