# Supplementary material for: Universal linear intensity transformations using spatially incoherent diffractive processors
Source: Light Sci Appl. 2023 Aug 15;12:195. doi: 10.1038/s41377-023-01234-y (PMC10427714; doi:10.1038/s41377-023-01234-y)
Supplement: Supplementary file 1 — Supplementary Information [file 41377_2023_1234_MOESM1_ESM.pdf]

Supplementary Information for

Universal Linear Intensity Transformations Using Spatially Incoherent Diffractive Processors

Md Sadman Sakib Rahman[1,2,3] mssr@ucla.edu
Xilin Yang[1,2,3] mikexlyang@ucla.edu
Jingxi Li[1,2,3] jxlli@ucla.edu
Bijie Bai[1,2,3] baibijie@ucla.edu
Aydogan Ozcan[1,2,3,*] ozcan@ucla.edu

[1]Electrical and Computer Engineering Department, University of California, Los Angeles, CA, 90095, USA
[2]Bioengineering Department, University of California, Los Angeles, CA, 90095, USA
[3]California NanoSystems Institute (CNSI), University of California, Los Angeles, CA, 90095, USA
[*]Corresponding author: ozcan@ucla.edu

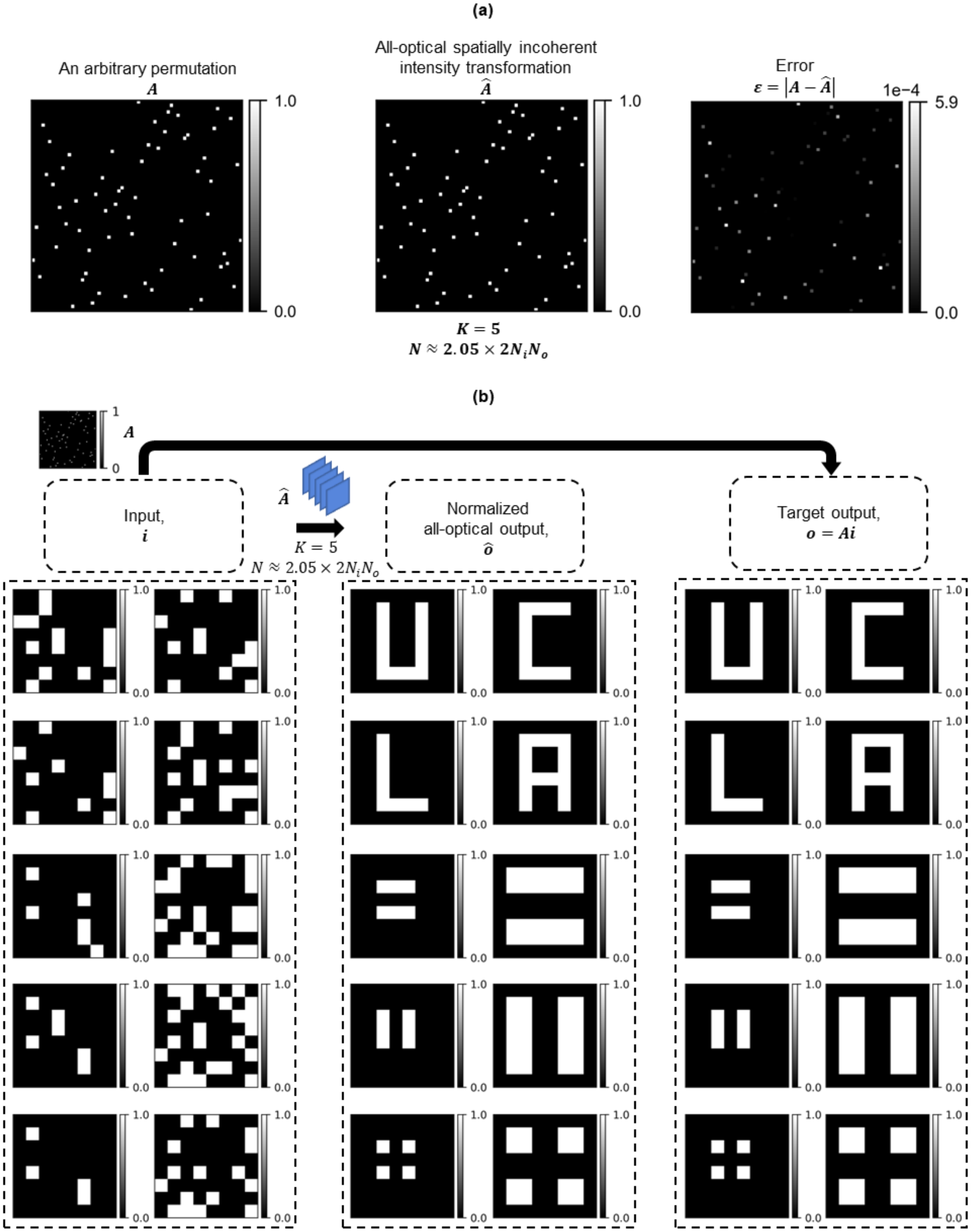

Fig. S1: Approximation of an arbitrary permutation $\boldsymbol{A}$ of intensity, under spatially incoherent illumination, by a diffractive network ($K = 5$, $N = 5 \times 58^2$) trained using the indirect approach. (a) The target transformation $\boldsymbol{A}$, the all-optical intensity transformation $\widehat{\boldsymbol{A}}$ performed by the diffractive network

and the error matrix $\boldsymbol{\varepsilon} = \left|\boldsymbol{A} - \widehat{\boldsymbol{A}}\right|$. Here $|\cdot|$ denotes elementwise operation. (b) All-optical linear intensity transformation of test patterns $\boldsymbol{i}$ (performed by the diffractive network) compared against the ground truth for various structured intensity patterns such as the letters U, C, L, and A, as well as closely separated line pairs and points.

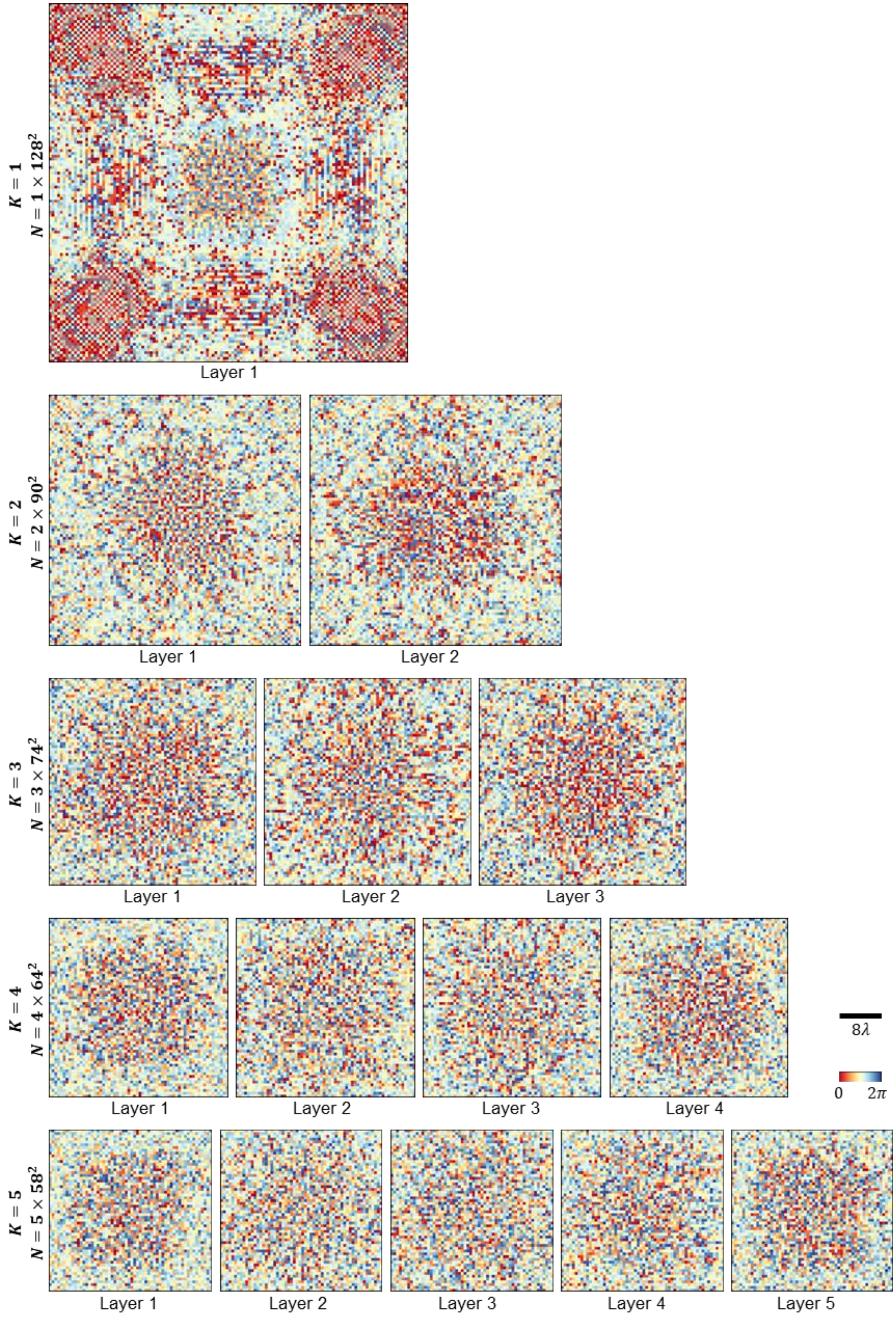

Fig. S2: Diffractive layer phase profiles for different designs with approximately the same number N of diffractive features distributed over K diffractive surfaces, for different K values.

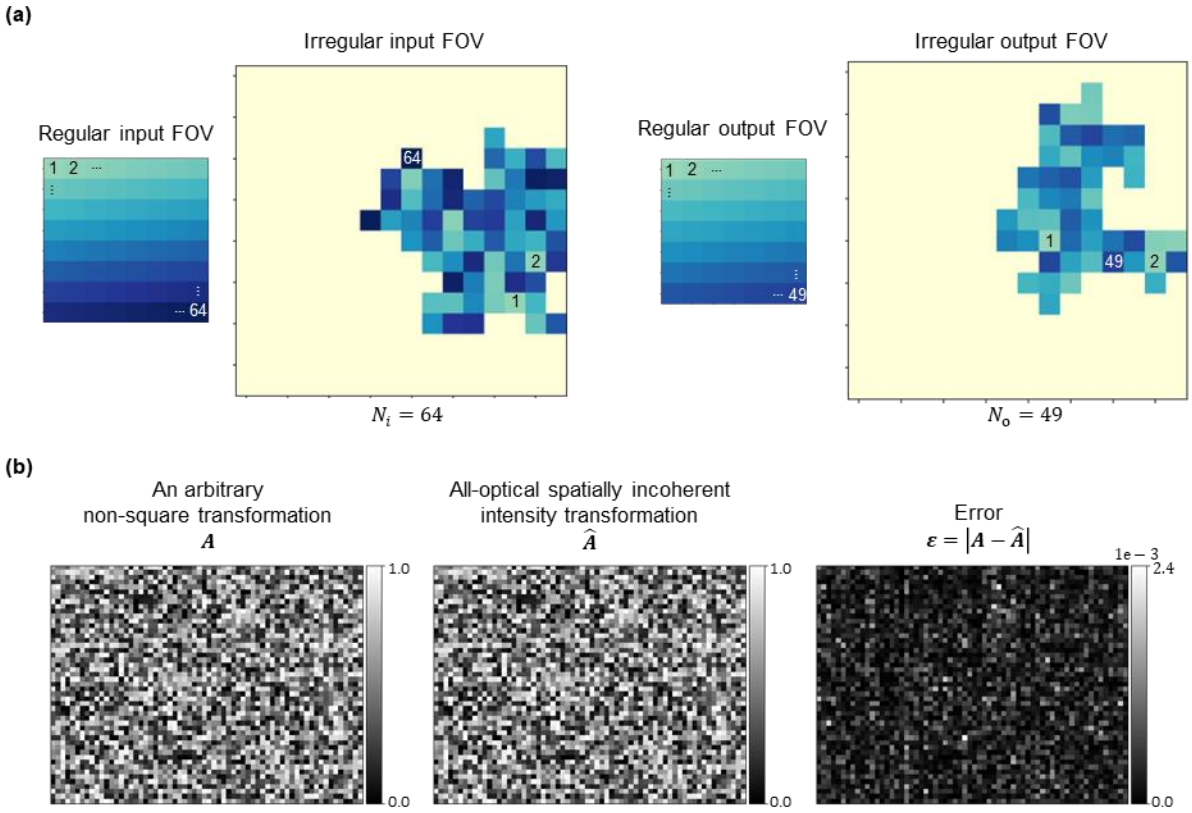

Fig. S3: Approximation of a non-square intensity linear transformation with a diffractive optical network under spatially incoherent illumination, trained using the indirect design approach. (a) We employed irregular distributions of $N_i = 64$ and $N_o = 49$ pixels on the input and the output FOVs, respectively; color encodes the indices of the input/output pixels. (b) The target transformation $\boldsymbol{A}$, the all-optical intensity transformation $\widehat{\boldsymbol{A}}$ performed by the trained diffractive network and the error matrix $\boldsymbol{\varepsilon} = |\boldsymbol{A} - \widehat{\boldsymbol{A}}|$ are shown. Here $|\cdot|$ denotes elementwise operation.

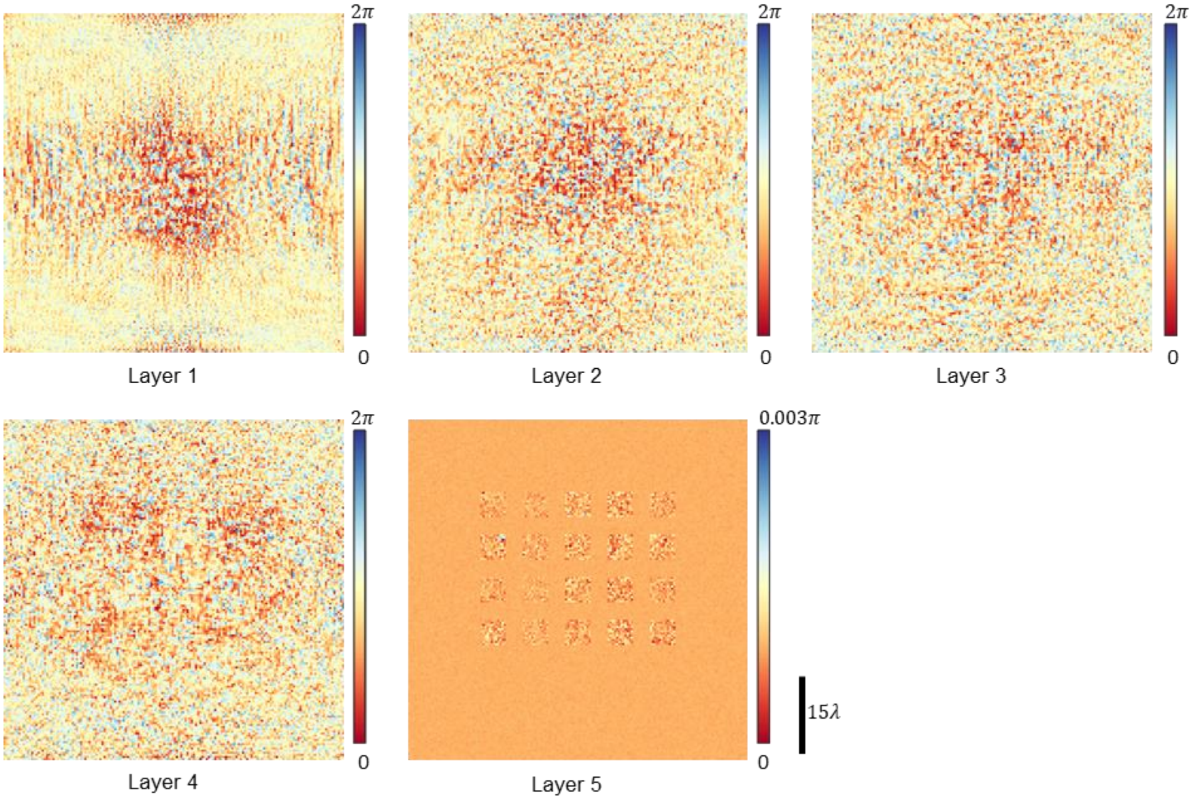

Fig. S4: Diffractive layer phase profiles for the spatially incoherent diffractive network classifier reported in Fig. 16 of the main text.

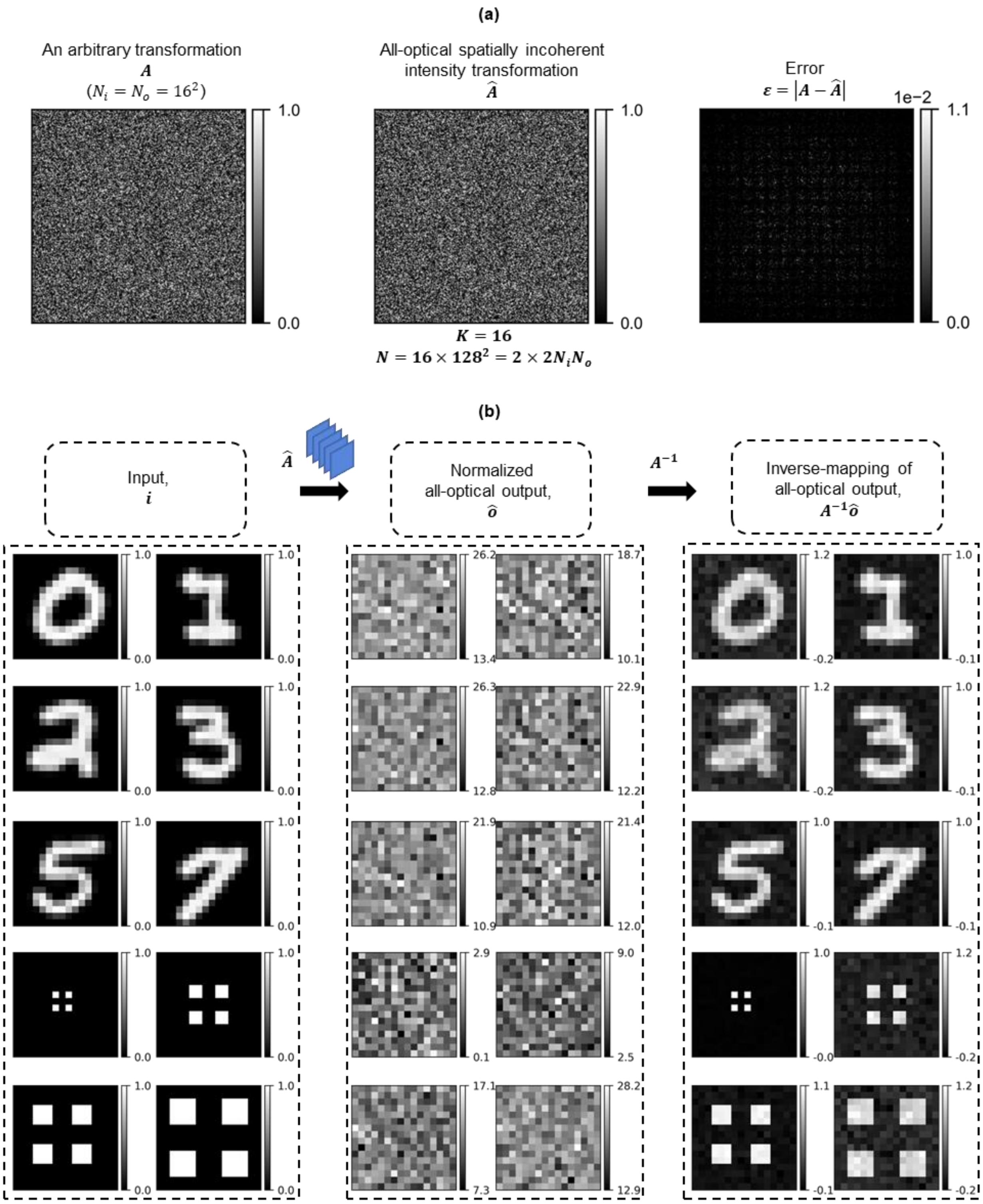

Fig. S5: Approximation of an arbitrary $16^2 \times 16^2$ linear transformation ($\boldsymbol{A}$) of intensity, under spatially incoherent illumination, by a diffractive network ($K = 16$, $N = 16 \times 128^2$) trained using the PSF-based data-free design approach. For this design, the size of the intensity pixels is diffraction-limited, i.e., $\sim\lambda/2$. (a) The target transformation $\boldsymbol{A}$, the all-optical intensity transformation $\widehat{\boldsymbol{A}}$ performed by the

trained diffractive network and the error matrix $\boldsymbol{\varepsilon} = \left|\boldsymbol{A} - \widehat{\boldsymbol{A}}\right|$. Here $|\cdot|$ denotes elementwise operation. (b) All-optical linear transformation of different test intensity patterns by the trained diffractive network, together with the patterns resulting from the numerical inverse mapping of the all-optical outputs through multiplication by $\boldsymbol{A}^{-\mathbf{1}}$. The all-optical output intensities are simulated using $N_{\varphi,te} = 640{,}000$.
